# Gene Networks Underlying Convergent and Pleiotropic Phenotypes in a Large and Systematically-Phenotyped Cohort with Heterogeneous Developmental Disorders

**DOI:** 10.1371/journal.pgen.1005012

**Published:** 2015-03-17

**Authors:** Tallulah Andrews, Stephen Meader, Anneke Vulto-van Silfhout, Avigail Taylor, Julia Steinberg, Jayne Hehir-Kwa, Rolph Pfundt, Nicole de Leeuw, Bert B. A. de Vries, Caleb Webber

**Affiliations:** 1 MRC Functional Genomics Unit, Department of Physiology, Anatomy and Genetics, University of Oxford, Oxford, United Kingdom; 2 Department of Human Genetics, Radboud University Medical Center, Nijmegen, the Netherlands; 3 Wellcome Trust Centre for Human Genetics, University of Oxford, Oxford, United Kingdom; Georgia Institute of Technology, United States of America

## Abstract

Readily-accessible and standardised capture of genotypic variation has revolutionised our understanding of the genetic contribution to disease. Unfortunately, the corresponding systematic capture of patient phenotypic variation needed to fully interpret the impact of genetic variation has lagged far behind. Exploiting deep and systematic phenotyping of a cohort of 197 patients presenting with heterogeneous developmental disorders and whose genomes harbour *de novo* CNVs, we systematically applied a range of commonly-used functional genomics approaches to identify the underlying molecular perturbations and their phenotypic impact. Grouping patients into 408 non-exclusive patient-phenotype groups, we identified a functional association amongst the genes disrupted in 209 (51%) groups. We find evidence for a significant number of molecular interactions amongst the association-contributing genes, including a single highly-interconnected network disrupted in 20% of patients with intellectual disability, and show using microcephaly how these molecular networks can be used as baits to identify additional members whose genes are variant in other patients with the same phenotype. Exploiting the systematic phenotyping of this cohort, we observe phenotypic concordance amongst patients whose variant genes contribute to the same functional association but note that (i) this relationship shows significant variation across the different approaches used to infer a commonly perturbed molecular pathway, and (ii) that the phenotypic similarities detected amongst patients who share the same inferred pathway perturbation result from these patients sharing many distinct phenotypes, rather than sharing a more specific phenotype, inferring that these pathways are best characterized by their pleiotropic effects.

## Author Summary

Developmental disorders occur in ∼3% of live births, and exhibit a broad range of abnormalities including: intellectual disability, autism, heart defects, and other neurological and morphological problems. Often, patients are grouped into genetic syndromes which are defined by a specific set of mutations and a common set of abnormalities. However, many mutations are unique to a single patient and many patients present a range of abnormalities which do not fit one of the recognized genetic syndromes, making diagnosis difficult. Using a dataset of 197 patients with systematically described abnormalities, we identified molecular pathways whose disruption was associated with specific abnormalities among many patients. Importantly, patients with mutations in the same pathway often exhibited similar co-morbid symptoms and thus the commonly disrupted pathway appeared responsible for the broad range of shared abnormalities amongst these patients. These findings support the general concept that patients with mutations in distinct genes could be etiologically grouped together through the common pathway that these mutated genes participate in, with a view to improving diagnoses, prognoses and therapeutic outcomes.

## Introduction

Developmental disorders and congenital abnormalities affect 3% of births, and represent an extremely heterogeneous group of disorders including intellectual disability, autism, and developmental delay, along a diverse range of structural and morphological defects[1]. The epidemiology of these heterogeneous disorders strongly implicates an underlying genetic etiology, with many patients possessing an increased burden of copy number variants (CNVs; regions of the genome > 1Kb that are deleted or duplicated). CNV screens now routinely included in primary diagnostics[2] with exome analyses expected to grow in use as the cost falls [3,4]. However, the heterogeneity in patient phenotypes is similarly reflected in the underlying genetic variation making it difficult to pin-point those particular genes whose mutation contributes to the phenotype. The field of disease genomics has, in particular, heralded two developments to address heterogeneous and multigenic disorders, namely (i) deep and systematically-defined patient phenotypes[5,6] and (ii) pathway analysis approaches[7,8].

For genetically heterogeneous disorders, the power of a cohort of patients to identify a shared pathoetiology may be diminished by the presence of multiple etiologies, each etiology contributing non-exclusively to different aspects of the phenotypic heterogeneity. Accordingly, it is often advantageous to analyse more phenotypically-similar subgroups, assuming that these subgroups would be enriched for a particular etiology[9]. Indeed, the observation by clinicians of marked phenotypic similarities across a broad range of features for a particular subgroup of patients has enabled the identification of the genetic causes of many syndromes; for examples see[10]. To enable large-scale, systematic and automated patient phenotypic comparisons, phenotype ontologies, such as the Human Phenotype Ontology (HPO)[11,12], have been developed. These ontologies consist of thousands of predefined phenotypic terms arranged in a hierarchy in which more specific child terms are organised underneath broader parent terms; for example, a patient ascribed the more specific phenotype “abnormality of the retina” would also recursively inherit any overarching phenotypic terms such as “abnormality of the eye”. Once these phenotypic terms have been rigorously assigned to large numbers of patients, ontologies such as the HPO enable various degrees of phenotypically-similar subgroups of patients to be systematically and objectively constructed, permitting the search for shared pathoetiologies at many levels of phenotypic homogeneity. A second challenge for large-scale analyses of patient phenotypes is that often only the presence of phenotypes is recorded without confirmation that unrecorded phenotypes had been considered. This forces the dangerous assumption that the absence of evidence for the presence of a given phenotype is evidence of absence of that phenotype. Many non-obvious and non-superficial genotype/phenotype relationships may be missed unless the presence/absence of phenotypes are explicitly determined and, in particular, pleiotropy will likely be under-reported[6].

Complementing patient phenotype subgrouping, pathway analysis approaches rely on the observation that disruptions to different genes that operate within the same pathway often produce similar phenotypes[9]. Thus, the analysis of patients that share a common phenotype, regardless of whether they can be classed into an over-arching disorder, may yield insights into commonly disrupted pathways underlying that phenotype and contribute to the spectrum of presentations in each patient. This approach is well suited to the study of multigenic disorders as pathway approaches gain power from coverage of the pathway, rather than recurrent hits to the same gene. One approach to identifying commonly disrupted pathways amongst distributed variants is to employ “functional enrichment” approaches[13]. This approach proposes that the genes affected by dispersed variants that act within a common pathway are likely to share other functional or biological characteristics, such as being annotated to a particular biological process[14,15], exhibiting a particular expression pattern[16], expressing protein products that interact with each another[17] and/or presenting a shared phenotype upon their orthologue’s disruption in a model organism[15,18,19]. Such collections of gene annotations vary both in terms of their rates of false positives, especially when formed from high-throughput experiments or computational-inference, and false negatives, for example where gene coverage is poor[20–22]. Thus, different annotation types may be combined to increase confidence that genes are functionally concordant[23,24].

In this study, we tested the paradigm that pathway analysis approaches applied to a large and systematically-phenotyped cohort that present heterogeneous developmental disorders can detect common molecular pathologies and the extent to which these inferred common etiopathologies confer common phenotypic presentations. Focusing on 197 patients that possessed *de novo* CNVs smaller than 5 Mb, we systematically grouped them according to the structure of the HPO and applied a range of complementary functional enrichment approaches that converged on numerous molecular pathways underlying a range of phenotypes. These gene networks were deemed biologically coherent through enrichments of direct molecular interactions, and we provide an exemplar as to how they can be used as “baits” to identify genes disrupted in other cohorts that participate in the same network. Finally, we show that patients whose variants contribute to the same functional enrichments are significantly more phenotypically-similar overall and results primarily from pleiotropic effects arising from the perturbation of the same inferred network, but that this similarity varies with the enrichment approach employed.

## Results

### Summary of data

We sought to employ common functional enrichment and pathway analysis to a large cohort of deeply and systematically-phenotyped patients presenting with heterogeneous developmental disorders, in order to identify the genes and molecular pathways underlying these phenotypes and to investigate the phenotypic similarity among patients whose variants affect genes detected to lie in the same pathways. We focused on sporadic patients possessing *de novo* CNV events as it is often the case that the *de novo* CNV mutation is consequential, and thus copy change of one or more of the genes disrupted by the CNV is responsible for the phenotypes observed^2; 10^. Furthermore, we excluded patients with CNVs >5Mb, as the large numbers of genes affected make it substantially more challenging to identify specific functional enrichments. Through our integrative approach, we sought to identify the genes and pathways underlying each phenotype presented by members of the cohort.

A cohort of 4297 patients was collected by the Radboud University Medical Centre, Nijmegen, the Netherlands. All patients were diagnosed with intellectual disability, developmental delay and/or congenital abnormalities. Each patient was deeply phenotyped by clinicians, using terms from the Human Phenotype Ontology (HPO) to describe their clinical abnormalities. The HPO contains over 10,000 terms for clinical phenotypic abnormalities, which relate to one another through a hierarchical structure, with less specific parent terms, covering more specific child terms. All parent (more general) phenotypic terms were additionally assigned to patients based on their clinically-assigned phenotypes. Central to the analyses performed in this study, these patients were systematically phenotyped and thus each patient was considered for the same phenotypes as every other, enabling accurate comparison of patient phenotypic similarity.

Of 1663 rare CNVs observed within at least one patient in the cohort, 437 were identified as *de novo*. Filtering our patients to include those that had only a *de novo* CNV shorter than 5Mb left 197 patients remaining (see **Materials and Methods**). Of these, 154 patients (78%) had intellectual disability of varying severity, 80 were diagnosed with developmental delay, 54 with growth abnormalities, and 37 with autistic spectrum disorder (ASD). These 197 patients possessed 826 distinct HPO phenotypes (individuals possessing 2–182 phenotypes, including parental terms), while the median number of phenotypes per patient increased to 31. There were 219 *de novo* CNVs remaining across the 197 filtered patients, with a median size of 1.37 Mb. Of these, 82 were duplication, or gain, CNVs, while the remaining 137 were deletion, or loss, CNVs. Gains and Losses had a median size of 1.36Mb and 1.40Mb respectively. 2907 unique genes were affected across all 197 patients, ranging from 0–190 genes per patient, with a median 95 gene disrupted per patient.

### Inferring molecular pathways

We employed a multifaceted functional genomics approach to analyzing the genes disrupted in the cohort. In turn, we investigated each set of genes found to be affected by *de novo* CNVs in patients that shared a specific HPO phenotype, where patients sharing the phenotype numbered 3 or more (408 patient-phenotype groups). For each phenotype, we employed a four-way functional genomics analysis, to identify functional associations between genes disrupted in these particular patient-phenotype groups which could represent a disrupted biological process that underlies the shared phenotype. Firstly, we employed a Gene Ontology (GO) analysis[25], in order to determine whether or not genes disrupted with each phenotype were associated with any particular GO terms, using a whole genome background as a comparator. The second method applied was to similarly determine enrichments among disrupted genes using pathway annotations from the Kyoto Encyclopedia of Genes and Genomes (KEGG) [26]. As a third method, we examined the abnormal phenotypes observed from gene disruption events (“knockouts”) in mouse [18,27]. For this, we asked whether the unique mouse orthologues of genes affected by these CNVs yield particular phenotypes when disrupted. Finally, our fourth method examined whether or not genes from patients’ CNVs clustered together in a gene co-expression network. Given these patients predominantly neurological phenotypes, we used the BrainSpan dataset which measured the spatiotemporal expression of genes across 16 brain regions and at 6 developmental time points (see **Materials and Methods**).

**Fig 1:**
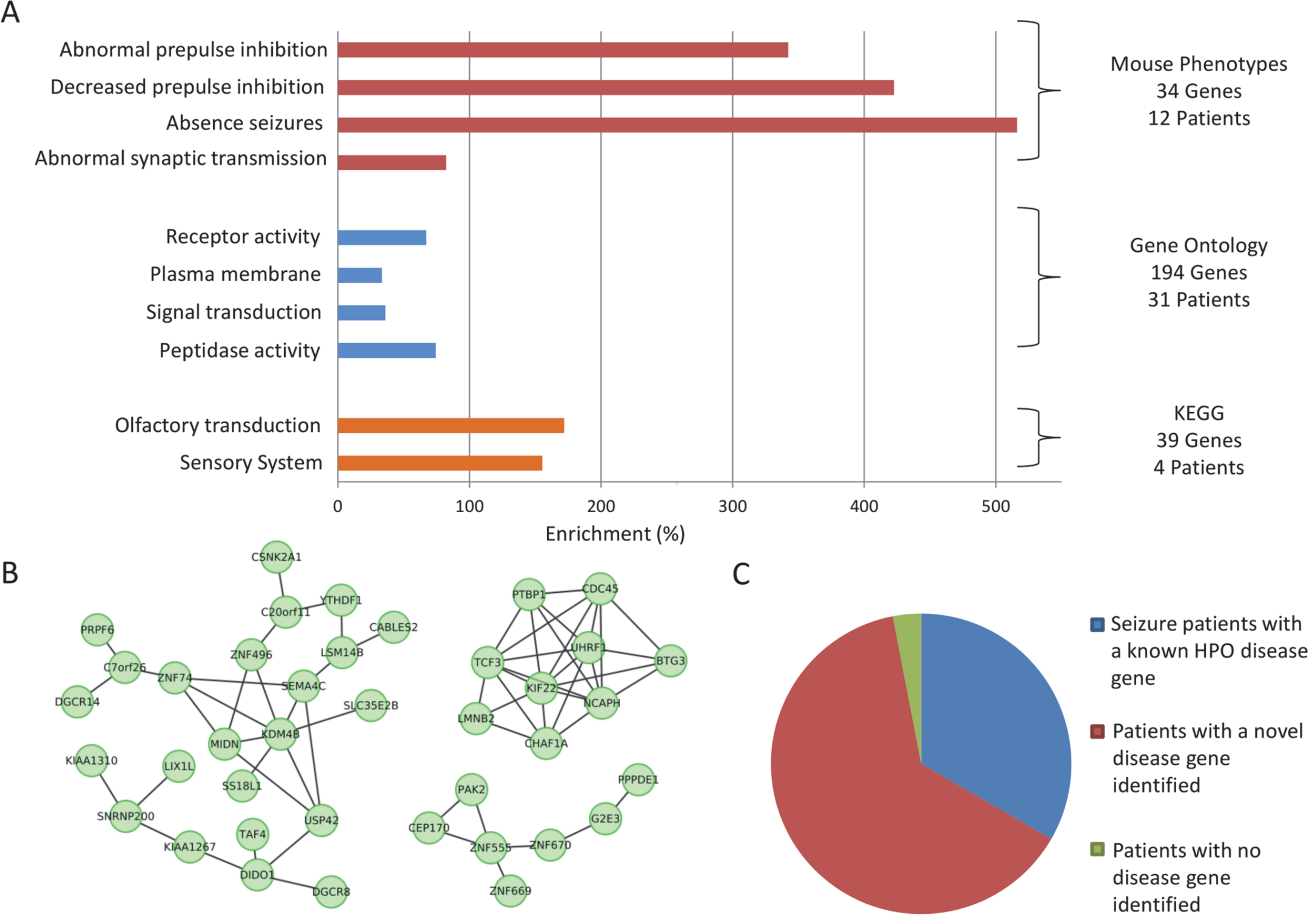
Functional genomics enrichments significantly enriched in genes affected by *de novo* CNVs in 33 patients presenting with seizures. **(A)** Significant functional genomics enrichments. Many of these functions have links to seizures or associated phenomena (synaptic deficits, receptor signaling, gustatory aura[73]) but also to regions prone to copy number variation[74]. **(B)** Genes disrupted by short CNVs in patients were also observed to cluster significantly in a brain-specific gene co-expression network. Here we display the strongest clusters (*r >* 0.92 for all co-expression similarities) of genes from seizure patients from this network. **(C)** Overall, the functional enrichments identified known (HPO-defined) seizure genes for 11 of the 33 patients, and proposed causal genes for 21 of the remaining 22 patients.

**Fig 2:**
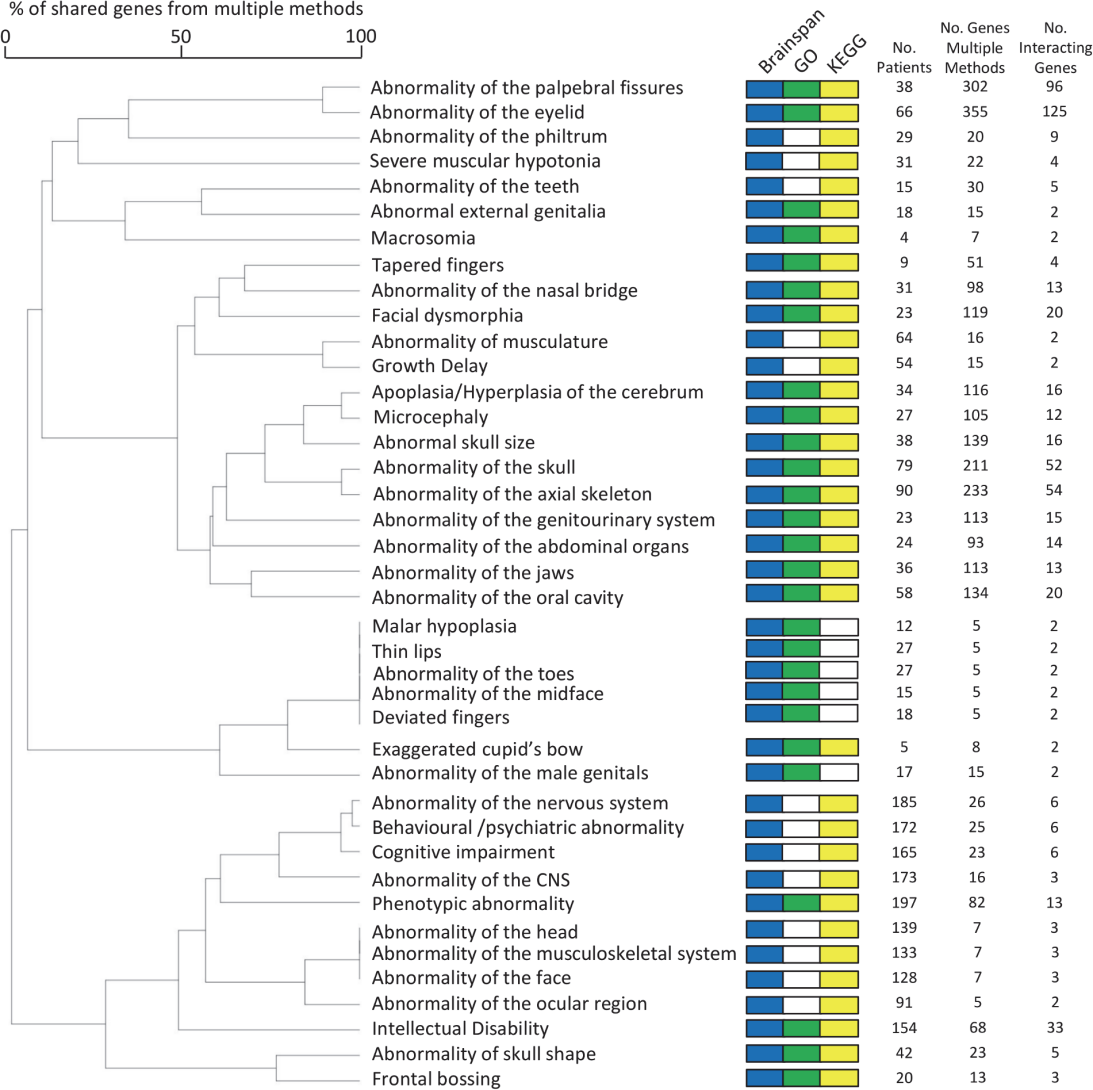
Forty non-exclusive patient groups, each group’s patients sharing the same HPO term, amongst whom individual copy number variant candidate genes were each recurrently identified by multiple functional genomics methods and whose recurrently-identified candidate genes demonstrated a significant number of protein-protein interactions. The dendrogram displays the relationship between categories based upon the number of candidate genes identified by multiple methods that are shared between the phenotype-group patients. Categories are marked if there were significant enrichments using clustering in a gene expression network (Blue), GO (Green) or KEGG (yellow). No phenotype-grouped patients with candidate genes meeting these criteria were identified use mouse KO phenotype (MGI) associations.

Each of these functional association approaches was applied to each of 408 sets of genes disrupted by CNVs in patients presenting a particular phenotype (patient-phenotype groups). Genes variant in only two patient-phenotype groups were found to possess significant functional associations using all four of the methods applied, namely HP:0001250 (*Seizures*) (**Fig. 1**) and HP:0010864 (*Intellectual disability*, *Severe*). Significant enrichments using three of the methods were observed in a further 64 patient-phenotype groups, enrichments for two methods for 120 patient-phenotype groups, and for just one method in a further 143 groups. Of the four methods employed, enrichments of phenotypes from mouse-orthologue knockouts (MGI) gave the least number of significant results, identifying functional association among affected genes in only 12 phenotype groups including HP:0001250 (*Seizures*) and HP:0000717 (*Autism*) (**S1 Table**). While the MGI method identified fewest associations, the enriched terms were the most relevant to the particular HPO phenotypes. For example, for patients with *Seizures* we saw an enrichment of genes whose mouse orthologue knockouts present with, amongst others (**Fig. 1**), *Absence seizures* (MP:0003216; 6.2-fold enrichment; *p* = 3 x 10^–4^). Similarly, in patients with HP:0010864 (*Intellectual Disability*, *Severe*) we see an enrichment of mouse synaptic phenotypes such as *Abnormal synaptic transmission* (MP:0003635; 3.3-fold enrichment; *p* = 2.0x10^–5^). This was in contrast with the results for *Intellectual Disability*, *mild* (HP:0001256) which was significantly enriched in learning phenotypes in mouse, such as *abnormal associative learning* (MP:0002062; *p* = 3.6x10^–6^). When these two subsets of patients were combined under the more general term of *Intellectual Disability* (HP:0001249) no mouse phenotypes were significantly associated but many signalling pathways described in KEGG were significantly enriched among the patients, including the *MAPK signalling pathway*, and *Neurotrophin signalling pathway*. Other methods provided a larger number of significant results, with enrichments of GO terms found for each of 121 human phenotypes; however these enrichments were generally smaller, and involve less specific categories than those observed using mouse phenotypes (S1 and S2 **Tables, S1 Fig.**). Additionally, affected genes within each of 189 phenotype groups were associated with particular KEGG pathways (**S3 Table**), while genes affected within each of 262 phenotype groups showed similarities in their brain spatiotemporal expression patterns, clustering significantly in the BrainSpan expression network (**S4 Table**). For 186 of 408 patient-phenotype groups, we found functional associations using multiple methods, and of these 177 (95%) phenotype groups identified the same genes using multiple methods, with the number of genes repeatedly identified for these groups ranging from 1 to 355 (**Fig. 2, S5 Table**).

A central tenet of pathway approaches is that a common phenotype can result from the perturbation of different genes that act within a common molecular pathway. We sought to validate that these functional association approaches were identifying commonly perturbed molecular pathways through protein-protein interactions, arguing that the protein products of genes involved in the same molecular pathway are more likely to directly interact with one another. Considering each of the 177 sets of multiply detected candidate genes and employing the Dapple protein-protein interaction (PPI) network[28], we found that 65 (37%) phenotype-grouped candidate genes showed significant clustering of genes within the PPI network demonstrating that the candidate genes identified for many of these phenotypes work together within the same molecular pathways (**Fig. 2, S4 Fig.**)

**Fig 3:**
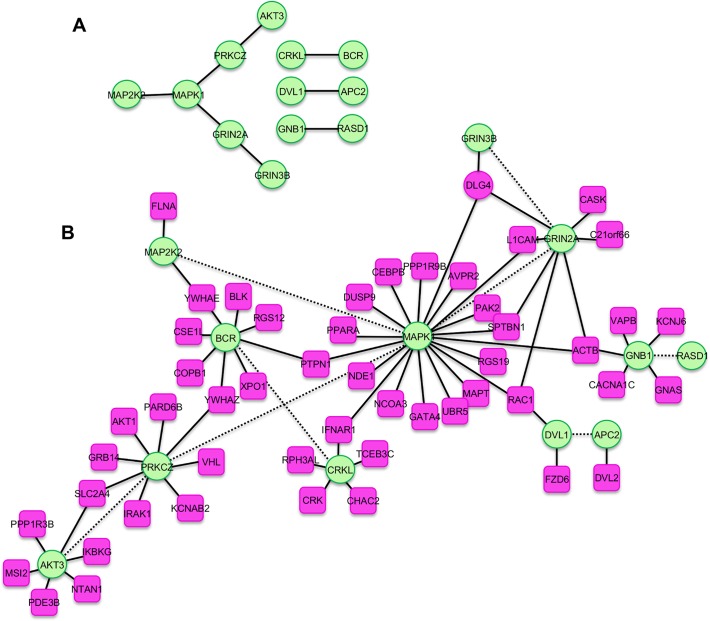
Molecular pathways serially identified among patients with microcephaly phenotypes in two large cohorts. **(A)** 12 copy variant genes drawn from 14 of 27 Nijmegen patients with Microcephaly that were identified using multiple functional genomics methods (KEGG, Gene Expression and GO) and cluster strongly (*p* = 0.04) in the Dapple protein-protein interaction network. **(B)** Genes (n = 51; Red)) that were copy number variant in 30 of 71 Decipher patients with Microcephaly were found to possess a significant number of interactions with the genes from panel A (Green) (*p* = 0.04), forming an extensive and intertwined microcephaly-associated molecular network.

To demonstrate the relevance of these phenotype-associated molecular networks beyond the cohort considered here, we looked for genes acting in the same molecular pathways that were affected by *de novo* CNVs in a second set of patients presenting with the same phenotype. Specifically, given each of the 65 PPI molecular networks perturbed by CNVs in Nijmegen patients presenting with the same specific phenotype, we asked whether the proteins encoded by genes found to be copy number changed in an additional cohort with the same phenotype also interacted within the same molecular networks. For this, we considered patients possessing *de novo* CNVs that were annotated within the DECIPHER database[29]. Although the DECIPHER patients have not been systematically phenotyped and their presentations denoted using the LDDB dysmorphology terms[30] rather than the HPO phenotypes, 15 LDDB terms used to describe their phenotypes could be mapped equivalently to 15 of the 65 HPO terms for which a PPI was identified in the NIJMEGEN patients (**S7 Table**). Of these 15 phenotypes, only for *Microcephaly* was there found to be a sufficient number of genes in both the NIJMEGEN-derived PPI network and DECIPHER patients CNVs to test for interactions. Nonetheless, after 13 DECIPHER patients whose variant genes participated in the NIJMEGEN-derived microcephaly PPI network, the genes variant in the remaining 58 DECIPHER patients with *Microcephaly* were found to interact with the NIJMEGEN microcephaly PPI network genes significantly more frequently than expected (*p* = 0.04; **Fig. 3B**), demonstrating recurrently hit phenotype-associated pathways. Most notably, whereas the NIJMEGEN-derived PPI molecular network alone is fractured into four unconnected sets of interacting genes, the interacting genes identified from the DECIPHER patients link three of these disparate groups to form a coherent molecular pathway (**Fig. 3B**). The PPI network perturbed in 14/27 (52%) of Nijmegen patients and perturbed in 13/71 DECIPHER patients with *Microcephaly* was able to identify genes interacting with the same network that were perturbed in an additional 30/71 (42%) of DECIPHER patients, implicating this network’s disruption in 57/98 (58%) of all microcephaly patients considered (**S6 and S7 Tables**).

### Phenotypic similarity between patients contributing to enrichments

Known developmental syndromes, particularly those involving ID, are frequently identified based upon shared phenotypic characteristics beyond ID, which may reflect the pleiotropic effects of a recurrently mutated gene [31–35]. Moving beyond a mutation in a single gene, we reasoned that if a functional enrichment identified among the copy number variant genes within a given set of patients identifies a common biological pathway perturbed within those patients, then the consequence of perturbing the same pathway may yield a similar set of phenotypes. To investigate this hypothesis, for each significant functional enrichment and the respective patient-phenotype group it was identified in (**S1–S4 Tables**), we subdivided the patients with that phenotype into those whose variant genes contribute to that specific functional enrichment (“contributing patients”) and those patients whose copy number variant genes do not contribute (“non-contributing patients”). Exploiting the consistent and structured phenotyping of the cohort, we then compared the pairwise phenotypic similarity amongst contributing patients to the pairwise similarity between contributing and non-contributing patients (**S5 Fig.**).

**Fig 4:**
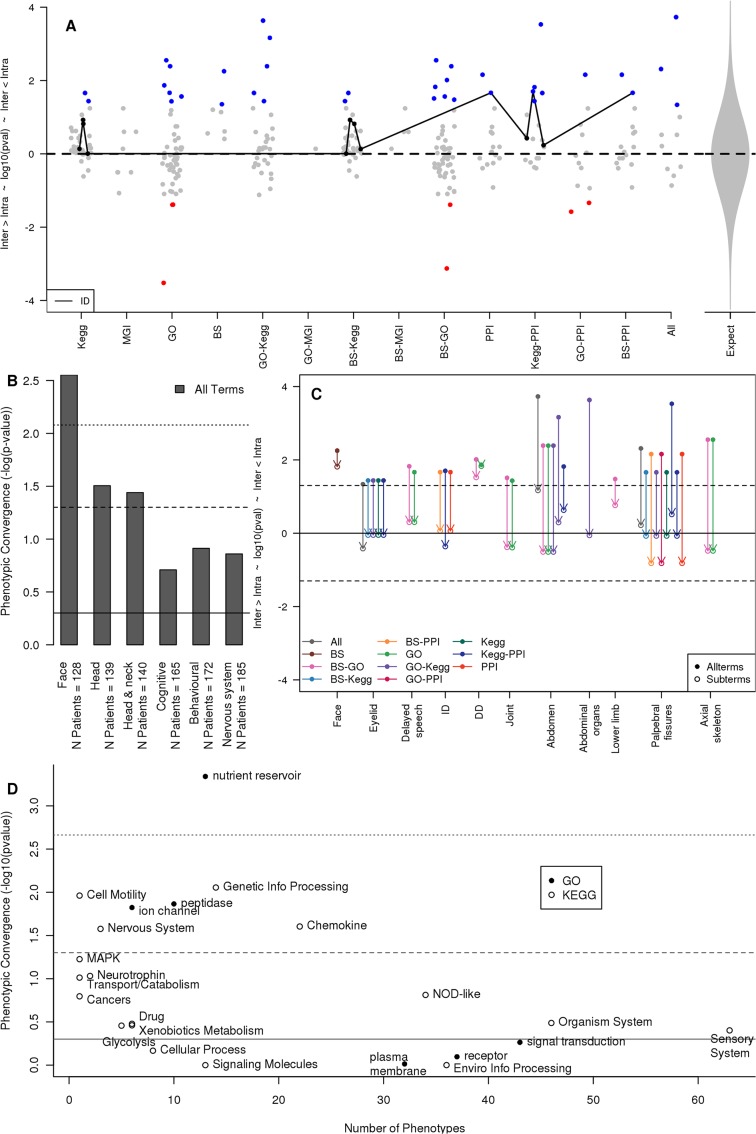
Phenotypic concordances amongst patients whose copy number variant genes contribute to the same functional associations and molecular pathways. **(A)** Overall, patients with genes that contribute to the same functional association are phenotypically similar (*p* = 1 x 10^–4^). The Y-axis gives the significance of the overall phenotypic similarity amongst patients within a patient-phenotype group whose variant genes contribute to a functional association (Intra) as compared to those patients in the same phenotype group who do not contribute (Inter), with higher values indicating increasing relative similarity amongst association-contributing patients. Each point represents a single significant patient-phenotype group association, while the methods used to identify the association are shown on the X-axis (KEGG, MGI mouse KO phenotypes, GO, BS BrainSpan gene co-expression). Combinations of methods (e.g. GO-KEGG) illustrate the relative phenotypic similarity amongst patients possessing copy variant genes that individually contribute to multiple functional associations (see **Results**). “PPI” values are those among patients contributing the interacting molecular networks identified in **Fig. 2** (see **Results**). Dots coloured blue or red indicate nominally significantly phenotypic similarity or dissimilarity, respectively. The black line connects all enrichments associated with the intellectual disability (ID) patient-phenotype group. **(B)** BrainSpan (BS) was the only pathway-resource to consistently identify phenotypically similar subgroups through a shared molecular association. Detail on the phenotypic similarities shown in Panel A. Solid line: p = 0.5, dashed line: p = 0.05, dotted line: p = 0.007 conferring significance after a Bonferroni correction. **(C)** The significant phenotypic similarities amongst patients who contribute to the same functional association are not derived from these patients presenting more specific subphenotypes of the original phenotype. Y-axis as in panel A. For all nominally significant enrichments in panel A (top, solid points) we recalculated the patient phenotypic similarities considering only child terms of the original HPO phenotype (open points connected to their respective solid point by an arrow). Points are grouped horizontally by HPO and coloured by enrichment-type. Solid line: *p* = 0.5, dashed line: *p* = 0.05. **(D)** In general, the fewer patient-phenotype groups that a functional enrichment term was associated with, the more phenotypically similar the patients associated with that functional term were. Patient-phenotype groups associated with the same KEGG pathway or GO term were combined and for each association the phenotypic similarity amongst those patients whose variant genes contributed to the given association was compared to those who did not contribute. Y-axis as in panel A. The number of patient-phenotype groups each functional association is associated with is given on the X-axis.

Overall, we found a significant excess of instances where contributing patients are more similar to each other than they are to non-contributing patients; p = 4 x 10^–4^, one-sided binomial test (**Fig. 4A**). However, this is highly variable between the different functional genomics resources used to identify the functional enrichment, as well as between phenotypes examined. Only patients whose copy number variant genes showed significantly co-ordinated brain expression patterns within the BrainSpan data were consistently found to be more similar in their overall phenotypes as compared to pairs of patients whose genes were not similarly co-ordinately expressed in the brain (**Fig. 4A** and **B**). Considering the remaining 3,871 patients without *de novo* CNVs, patients whose CNVs affected genes co-expressed within BrainSpan continued to show the most significant phenotypic similarity when the analysis was repeated considering the phenotypic similarity amongst patients whose inherited CNVs affected the previously-identified candidate pathway genes. This was also true for phenotypic comparisons involving patients whose CNVs affected genes related to the candidate pathways (have the same annotation or are co-expressed with or have a PPI with candidate pathway genes (See **Methods**)), but here patients whose CNVs affected novel genes with the same GO annotation also demonstrated phenotypic convergence (**S6 Fig.**). Furthermore, restricting the patient phenotype comparisons to those patients possessing copy number variant genes that contributed to enrichments identified by two different functional resources did not increase the proportion of cases where contributing patients were more similar to each other than to non-contributing patients (14% vs 13%, p >0.9, **Fig. 4A**).

For those phenotypic comparisons where significantly similar phenotypes were observed amongst patients contributing to a functional enrichment (p < 0.05, 2-sided Wilcox-rank-sum test; **Fig. 4A**, blue points), we considered whether the functional enrichment was segregating patients based on more fine-scale differences of the phenotype that was associated with the enrichment or whether it reflected co-morbid phenotypic characteristics distinct from the enrichment-associated phenotype. To examine this, we repeated the phenotype-similarity analysis including only the child terms (subterms) of the enrichment-associated phenotype term (**Fig. 4C**). In almost all cases, the distribution of subterms was indistinguishable between patients contributing to the pathway enrichment and non-contributing patients, demonstrating that the phenotypic similarity between patients whose variant genes contribute to the same functional enrichment was produced by common co-morbidities of phenotypes distinct from the enrichment-associated phenotype. Taken together, our findings propose that the copy number variation of genes that contribute to those functional enrichments shared by patients who present with significantly similar phenotypes underlie the broad spectrum of phenotypes presented by these patients as a consequence of the perturbing the same inferred molecular pathway.

Finally, since the same KEGG and GO annotations were significantly associated with multiple human phenotypes, we combined patients from multiple patient-phenotype sets (patients sharing a particular phenotype) where those phenotypes had been associated with the same GO or KEGG pathway (**Fig. 4D**). Three GO pathways were significantly associated with fewer than 15 human phenotypes (*ion channel*, *nutrient reservoir*, and *peptidase*); these identified subsets of patients who are significantly more similar to each other, whereas the remaining three GO pathways (*plasma membrane*, *receptor*, and *signal transduction*) were significantly associated with more than 30 human phenotypes and did not identify phenotypically similar patient subsets. Similarly the four KEGG pathways significantly associated with more than 30 human phenotypes failed to identify phenotypically similar patient subsets. However, only five of the thirteen KEGG pathways associated with fewer than 30 human phenotypes identified phenotypically similar patient subsets; these tended to be the more biologically plausible pathways (eg. *Cell motility*, *Nervous system*, and *Transport and Catabolism*). The eight which failed to identify phenotypically similar patient subsets tended to be more general or unexpected pathways (eg. *Glycolysis*, *Drug*, and *Cancer*; **Fig. 4D**). Pathways significantly enriched in many human phenotypes may reflect biases in CNV occurrence, which have not been completely eliminated by removing genes found in control CNVs (see **Materials and Methods**).

## Discussion

**Fig 5:**
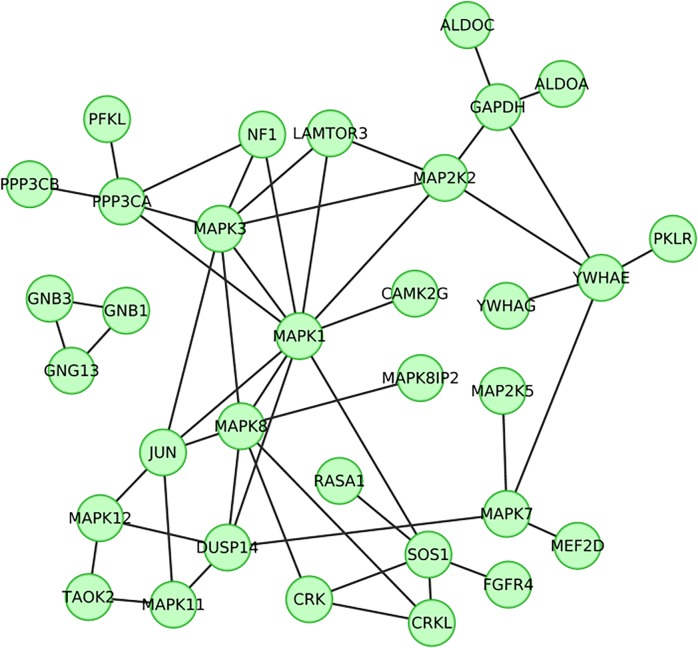
Clusters of 33 genes whose products have known protein-protein interactions copy changed among 34 (22%) of 154 patients with intellectual disability. These genes were those identified using two or more methods (from KEGG, GO and Gene Expression clustering) and that were found to contribute to a significant enrichment of interactions identified by the Dapple protein-protein interaction network (*p* < 1x10^–4^).

This study represents the first systematic functional genomics analyses of a systematically and deeply phenotyped cohort of patients presenting with developmental disorders. By grouping patients on the presence of a common phenotype, as defined by a shared HPO term, and applying often-used functional enrichment approaches to the genes affected by those patients’ *de novo* CNVs, we identified functional enrichments for 329 (81%) of 408 patient-phenotype groups (**S1–S4 Tables**). For 177 patient-phenotype groups the same genes were identified using more than one approach; and for 65 (37%) of these we found evidence for a significant number of molecular interactions between genes supporting shared molecular pathoetiologies (**Fig. 2, 3** and **5; S4 Fig.** and **S6 Table**). The generality of these pathways was further demonstrated by the ability of the microcephaly PPI network to identify additional pathway members mutated in another cohort with a similar phenotype (**Fig. 3**). Exploiting the “evidence of absence” of a phenotype among these patients, we were able to test the phenotypic convergence amongst patients whose variant genes contribute to the same functional enrichment. Overall, we find that there is significant phenotypic convergence providing general support for the “same perturbed molecular pathway, similar resulting phenotype” paradigm, but we note (i) that this relationship shows significant variation across the different functional genomics resources used to infer a commonly perturbed molecular pathway among a group of patients, and (ii) that these phenotypic convergences result from these patients sharing many distinct phenotypes, rather than sharing a more specific phenotype, suggesting that these pathways are best characterised by their pleiotropic effects (**Fig. 4**).

Phenotypic convergence amongst patients whose variant gene contributed variant gene contributed to inferred (GO, expression) and/or determined (KEGG, PPI) molecular pathways was identified from the shared spectrum of distinct phenotypes presented by these patients, indicating common pleiotropic effects arising from the disruption of the inferred molecular pathway. Patients with the same perturbed pathway did not form a more specific phenotypic-subgroup, based upon sharing more specific features (subterms), within the whole group pf patients presenting that phenotype (**Fig. 4C**). Inevitably, this may in part be a consequence of the phenotypic resolution captured for these patients, but we note that most patient phenotypic clustering based on subterms performed poorly including the more phenotypically-detailed “Abdomen” patient/pathway groups. Irrespectively, our finding of common pleiotropic effects arising from perturbing the same pathway strongly supports broad, systematic patient phenotyping to identify shared underlying molecular pathology [6,36].

Systematically combining large-scale molecular and phenotypic variation is at the heart of disease genomics. However, the limitations of attempts to correlate imposed categorisations of both gene function and patient phenotype are obvious; both depend on limits of experimental/diagnostic techniques, prevailing ideas of biologically- or clinically-relevant observations, and motivation to investigate a given gene or patient. Surprisingly, the functional enrichment resource that is arguably the most well-aligned with HPO phenotypes[37], namely the MGI mouse phenotypes, appeared to perform poorest in this study, both in detecting functional associations among variant genes (**S1 Table**) and in identifying phenotypically homogenous patient groups (**Fig. 4A**). Importantly, neither the literature-based pathways of the KEGG database nor GO-defined functionality delivered as many functional associations, nor identified as much phenotypic homogeneity within associations, as the most functionally/phenotypically-agnostic approach of correlated gene expression (BrainSpan; **Fig. 2** & **4B**). Despite the pleiotropic character of the phenotypic convergences, we found that a brain-specific gene expression set (BrainSpan) proved more effective than a body-wide expression set (GTEx; see **Materials and Methods**; **S2 Fig.**). This may reflect the preponderance of neurological phenotypes in the cohort and/or the value of BrainSpan’s longitudinal expression data. Regardless, these findings bode well for future studies as ever more detailed systematic gene expression maps are promised[38] (www.brain-map.org).

Despite current limitations in our ability to map phenotypes between different cohorts/standards, we demonstrated the utility of these networks. Using the *Microcephaly* PPI network as a “bait”, we were able to identify an additional 51 gene members of this interaction network copy changed amongst DECIPHER *Microcephaly* patients, and found this network perturbed in 58% of all microcephaly patients considered here (**Fig. 3**). The “bait” network (**Fig. 3A**) contained genes such as *AKT3*, considered the underlying cause for the microcephaly in patients with the 1q44 microdeletion syndrome[39], and *MAPK1*, involved in neurogenesis[40] and located within the distal 22q11 deletion frequently associated with microcephaly[41]. In addition, the extended network (**Fig. 3B**) contains several genes previously associated with an abnormal head circumference including *ACTB* in Baraitser-Winter syndrome (MIM #243310)[42], *AKT1* in Proteus syndrome (MIM #176920)[43], *CASK* in Mental retardation and microcephaly with pontine and cerebellar hypoplasia (MIM #300749)[44], and *NDE1* in microcephaly and lissencephaly[45,46]. Other genes within the combined network are involved in neuronal progenitor cell proliferation, a common mechanism underlying microcephaly[47](*RAC1*[48,49] and *GNB1*[50]) and neuronal development (*CEBPB*[51], *L1CAM*[52], *MAPT*[53], *PAK2*[54], *SPTBN1*[55], and *YWHAE*[56,57]).

The most common phenotype, *Intellectual disability* (ID), was observed in 78% of the cohort considered here, and thus is of particular interest. Within the *de novo* CNVs of the 154 patients presenting with ID, 68 potential candidate genes were identified using enrichments from at least two different methods (GO, KEGG, or BrainSpan expression). Of these 68 genes, the proteins expressed by 33 genes had known interactions with proteins expressed by other candidate genes, far more than would be expected by chance (*p* < 1x10^–4^), with 30 of these genes forming one single interacting cluster (**Fig. 5**). This cluster comprises of a selection of genes that have been associated previously with intellectual disability, such as *YWHAE* [58], *SOS1[59]*, and *MAP2K2* [60], and several whose function makes them likely candidates for involvement in ID. For example, *CAMK2* encodes a subunit of the Calcium/calmodulin-dependent protein kinase type II that is critical for regulation of synaptic plasticity[61]. This gene has been found to harbor a *de novo* mutation in a patient with severe intellectual disability[4], and an intronic SNP (rs11000787) which has been associated with memory performance[62]. In addition, *MEF2D* is a member of the myocyte enhancer factor-2 family of transcription factors that regulates neuronal development[63]. Mutations in another member of this family, *MEF2C*, have been observed previously in patients with severe ID[64]. Furthermore, ID patients whose CNVs perturb this network present significantly similar phenotypes as compared to other patients with ID (*p* = 0.016; **Fig. 4A**). The single interacting cluster of 30 genes in this network potentially explains 31/154 (20%) of patients with ID in the cohort (**Fig. 5**), and thus the nature of this molecular convergence warrants further study.

Finally, the 826 phenotypes present amongst patients in this cohort do not represent the full spectrum of human phenotypic variation (the HPO ontology alone contains 10,000 unique terms). Even the focused phenotyping of developmental abnormalities among the cohort considered here could be significantly enriched through detailed brain imaging, as well as longitudinal and more quantitative phenotyping[36]. Similarly, our focus only on those genes affected by *de novo* CNVs ignores the influence of the genetic background on phenotype[65]. Nonetheless, the systematic genotyping and phenotyping of these patients has in turn enabled systematic pathway-based genotype/phenotype approaches that identify extensive molecular networks that appear perturbed, often with pleiotropic consequences, thereby giving insight into the more rigorously genotyped and phenotyped future of genomic medicine. These pathways could be used to categorize patients with currently unknown developmental disorders and potentially identify new developmental syndromes.

## Materials and Methods

### Copy number variants

The patient data were previously described in detail in a manuscript by Vulto-van Silfout et al.[66], but shall be described here in brief. Four thousand two hundred and ninety seven patients with either intellectual disability, developmental delay, and/or multiple congenital abnormalities were recruited by the Radboud University Nijmegen Medical Centre. Each patient was phenotyped by clinicians using a uniform and standardised clinical form, classified using the Human Phenotype Ontology (HPO) terms[12]. More general HPO terms assigned to individuals were imputed from specific terms recorded. Of ∼10,000 possible HPO phenotypic terms covering the full spectrum of human phenotypic abnormalities, 1350 terms were assigned to one or more patients within the cohort. DNA samples were mainly taken via peripheral blood and analysed using the Affymetrix 250k *Nspl* SNP array platform (262,264 SNPs, 200 Kb resolution). CNVs were called where there were at least five or seven consecutive aberrant SNPs, for losses and gains, respectively. Where CNVs were observed, parental DNA was considered in order to determine whether CNVs were *de novo*, or to determine the mode of inheritance. We focused on the subset of 197 patients who possessed likely-causative *de novo* CNVs of <5Mb, amongst whom a total of 826 HPO phenotypic terms had been assigned.

To demonstrate the utility of the networks identified in this study (see **Results**), we selected 93 *de novo* CNVs possessed by 71 patients annotated with a *Microcephaly* (London Dysmorphology Database code: 32.08.05) phenotype that were recorded within the DECIPHER (DatabasE of Chromosomal Imbalance and Phenotype in Human using Ensembl Resources) database (**S7 Table)**. Merging overlapping (by at least 1bp) or bookended CNVs (losses and gains combined) yielded 76 CNV regions (CNVRs). From these 76 CNVRs, we removed 8 CNVRs (representing 13 patients) that affected genes that already participate in the *Microcephaly* network we had identified among the Nijmegen cohort (**Fig. 3**) and removed a further 12 CNVRs that did not affect genes annotated within the protein-protein interaction (PPI) database and thus could not be considered. Our final set of DECIPHER CNVs from *Microcephaly* patients contained 55 CNVRs, overlapping 606 genes that were represented in the PPI database (**S7 Table)**.

#### Ensembl gene IDs

Genomic co-ordinates of CNV regions were mapped from hg17 to hg18 using the USCS LiftOver tool (http://genome.ucsc.edu/cgi-bin/hgLiftOver) through the bedTools application (https://code.google.com/p/bedtools/). Gene annotations were taken from Ensembl using the Ensmart 54 database. Genes were considered to be completely overlapped if the entire Ensembl gene was within the CNV boundaries. Partial gene disruptions were those cases where a gene is not entirely overlapped by a CNV, but the CNV intersects with at least one exon in every transcript in the database. This method ensures that all coding transcripts of a gene are affected, not just a fraction of very long transcripts, and has been demonstrated to remove length biases associated with genes that show brain-specific expression patterns[13,17]. CNVs which did not affect all coding transcripts of a gene were excluded. As we were primarily interested in genes whose *de novo* copy change would be highly penetrant, we removed 5768 genes that had been observed as copy number changed in the same direction within the Shaikh *et al*. set, a control cohort that represents deleted and duplicated regions in individuals with no overt abnormalities[67].

### Functional enrichments

#### Mouse genome informatics (MGI) phenotypes

The phenotypes exhibited during published mouse gene model experiments and described according to the Mammalian Phenotype Ontology (MPO)[68] were obtained from the Mouse Genome Informatics (MGI; http://www.informatics.jax.org)[69]. For each patient, we mapped the disrupted genes to mouse genes using simple, unambiguous, 1:1 human:mouse gene orthology relationships as defined by MGI. For each set of patients annotated with a specific HPO term, we mapped the HPO term over to MPO, to identify the most relevant of 33 overarching categories within the MPO. For each of the mouse phenotypic terms within the relevant overarching category, we determined enrichments amongst the respective set of variant human genes, by comparing the frequency of variant genes associated each phenotype as compared to the frequency expected given the whole genome background using a hypergeometric test (False Discovery Rate (FDR) of less than 5%[70]). Underpowered and uninformative results were reduced by excluding phenotypes populated by less than 1% of the genes in the overarching category.

#### Gene ontology

Gene Ontology (GO) data were obtained from the Gene Ontology website (http://www.geneontology.org/). For a set of patients associated with a specific HPO phenotype, we looked for enrichments as compared to the genomic background using a hypergeometric test and an FDR of less than 5%.

#### KEGG

Gene annotation data was obtained from the Kyoto Encyclopedia of Genes and Genomes (KEGG; http://www.genome.jp/kegg/). For a set of patients associated with a specific HPO phenotype, we looked for enrichments as compared to the genomic background using a hypergeometric test and a FDR of less than 5%. The majority of the detected functional enrichments using MGI, GO or KEGG pass a more stringent Bonferroni correction.

#### Gene expression network

Normalized RNAseq gene expression data from was downloaded from BrainSpan (http://www.brainspan.org; 16 brain regions, 41 individuals aged from 8 weeks post-conception to 40 years). Genes with RPKM<1 in >95% of the samples were excluded and the expression correlation between each pair of remaining genes was calculated. A network was built with genes as nodes and edges between two genes weighted with their correlation coefficient *r*, considering only edges with weight *r*≥ 0.7 gave 13,953 unique genes with at least one edge. We confirmed our findings at *r*≥ 0.6 and *r*≥ 0.8, but found inconsistent and diminished results when *r*≥ 0.9 (**S7 Fig.**). We compared the strength of connections between genes in our test set (i.e. the sum of the correlation coefficients between the genes), as compared to genes randomly sampled from the co-expression network. We controlled for the number of edges associated with each gene (i.e. degree) by randomly sampling without replacement a maximum of 10,000 sets equal in gene number and that possessed the same gene degree distribution to determine significance.

Given our findings of pleiotropic effects associated with the perturbations of the inferred pathways we report here (see **Results**), we briefly examined employing a recently-released body-wide expression data, GTEx[38], instead of the brain-specific dataset, BrainSpan. A coexpression network was derived from the GTEx data using the correlation method used in[71] which accounts for missing data. However, we found that BrainSpan-derived molecular associations outperformed GTEx-derived association in the phenotype comparisons on which this decision would have been based, and thus retained the BrainSpan-derived co-expression approach (**S2 Fig.**).

#### Protein-protein interactions

All protein-protein interaction (PPI) data were obtained from the Dapple website (http://www.broadinstitute.org/mpg/dapple/dapple.php). We determined whether or not gene sets clustered within the protein-protein interaction network by comparing the number of interactions between test genes to randomly sampled gene sets, controlling for the number of degrees in the same manner as performed with the gene-expression network.

To investigate the connections between the genes within the Nijmegen *Microcephaly* (HP:0000252) PPI network and the genes variant in DECIPHER patients presenting with *Microcephaly* (**Fig. 3B**), we took the set of 12 genes participating in the NIJMEGEN-derived microcephaly PPI network (**Fig. 3A**) and asked whether they were more connected to the 606 genes (in the PPI database) affected by 55 CNVRs identified amongst the DECIPHER microcephaly patients (see **Data**; **S7 Table**), than to genes hit by 500 randomised sets of 55 CNVRs, matched in the number of contiguous genes that were present in the PPI database as the original set. Randomised regions were prohibited from containing any of the 12 genes participating in the NIJMEGEN-derived microcephaly PPI network.

### Phenotypic similarity

Phenotypic similarity between patients was calculated using the *Goodall3* measure [72]. The *Goodall3* measure gives a high weight to the shared presence of rare phenotypes and the shared absence of common phenotypes and was deemed more appropriate than other measures such as semantic similarity (**S3 Fig.**). For each pair of patients, the phenotypic similarity was calculated as the sum of the weighted similarity (*G*) of the presence/absence of each of all the phenotypes annotated to any of the 197 patients considered, where *G* is weight by the frequency (*f*_*i*_) of the phenotype in the patient population:

Gi={1-fi2ifiis present in both patients1−(1−fi)2ifiis present in neither patient0ifiis present in only one patient

For each of the significant functional enrichments, the group of patients sharing the respective HPO term was divided into those patients with variant genes participating in the enrichment (“contributing patients”) and those without (“non-contributing patients”). The significance of the difference between the phenotypic similarity amongst contributing patients and between contributing patients and non-contributing patients was evaluated using a two-sided Wilcox-rank-sum test (**S5 Fig.**). To ensure the test was well-powered, only those cases where there were at least 10 contributing patients and at least 10 non-contributing patients were considered.

#### Replicating phenotypic convergence

We replicated the phenotype analysis described above using inherited CNVs amongst those 3,871 patients who did not possess a *de novo* CNV. We reasoned that if the pathways identified above are indeed responsible for those patients’ phenotype then these same pathways could be used to identify the potentially pathogenic CNVs from the likely benign CNVs among the 1,043 inherited or unknown inheritance CNVs identified in these patients. We placed patients with the same specific phenotypic abnormality into three mutually exclusive groups: (1) ‘candidate pathways’, defined as those patients whose CNVs affect genes identified previously in the *de novo* CNV analyses above that contributed to significant functional enrichments; (2) ‘Extended pathways’, defined as those patients whose CNVs affect no gene included in the candidate pathways but that are nonetheless annotated with the same GO or KEGG function as previously associated functional enrichments or, for BrainSpan and the PPI, genes with a direct functional link to one of the genes in the functional enrichment; (3) ‘No pathway’, defined as those patients that possess either no CNVs or whose CNVs do not affect genes within previously associated pathways. Firstly, phenotypic similarity was calculated within the group of patients affecting candidate pathways as compared to the phenotypic similarity observed between the patients in this group and those within the combined group of patients affecting extended pathways or no pathway. Secondly, the phenotypic similarity was calculated within the group of patients whose CNVs affected the extended pathways as compared to the phenotypic similarity between patients within the extended pathway group and patients affecting no pathway.

## Supporting Information

S1 Fig Distribution of significant functional enrichments across phenotypes with different numbers of patients. (A) Average number of significant functional enrichments per phenotype. Error bars indicate 95% Confidence Intervals. (B) Total number of significant enrichments by data type: Gene Ontology (GO), Kyoto Encyclopaedia of Genes and Genomes (KEGG), mouse knockout phenotypes (MGI), BrainSpan gene co-expression (BS). (C) Number of significant annotations (GO, KEGG, or MGI) vs the number of patients with the respective HPO and the number of genes in the whole genome with the respective annotation.
(TIF)

S2 Fig Comparing GTEx and BrainSpan co-expression networks. **(A)** Pearson correlation between GTEx and BrainSpan co-expression networks (all edges with r > 0.5 in both networks). Each point is the average taken over 5000 edges. The Pearson correlation coefficient on the unbinned data is noted in the corner of the plot (r = 0.14); (**B**) Phenotypic similarity of subset of patients contributing to a significant GTEx co-expression network. Solid line is p = 0.5, dashed line is p = 0.05. Dark bars are using all phenotypes to calculate phenotypic similarity; light bars are using only the subterms of the original human phenotype to calculate phenotypic similarity.
(TIF)

S3 Fig Comparing Goodall similarity metric to semantic similarity (SS) for patient phenotype comparisons. While semantic similarity strongly weights the presence of a shared rare character, nothing is learned from the shared absence of a character. By comparison, the Goodall metric considers both the presence of shared rare character and the absence of a common shared character towards the overall similarity. The Goodall metric is thus more suitable where both the presence and absence of phenotypes are known, as is the case here with the systematically phenotyped Nijmegen cohort.
(TIF)

S4 Fig Protein-Protein Interaction (PPI)-defined molecular networks formed from genes copy number changed in patients who share the specified phenotype. The genes considered for these networks were those that were identified from multiple functional genomics/pathways approaches (GO, KEGG, BrainSpan, or MGI). See **Fig. 2**. Only those 14 PPI networks identified that contain a minimum of 5 genes are shown.
(PDF)

S5 Fig Testing phenotypic convergence. Each set of patients sharing a given phenotype was subdivided using each significant functional enrichment associated with that phenotype, eg. the KEGG Neurotrophin pathway, which was significantly enriched among CNV genes in patients with intellectual disability (ID). One group, ‘contributing patients’, contained all patients with the phenotype (ie. ID) whose CNV affected genes contributing to the functional enrichment (ie. belong to the Neurotrophin pathway); the other patients with the phenotype were placed in the ‘non-contributing’ group. Then the distributions of pair-wise patient phenotypic similarity within the ‘contributing’ group and between groups was calculated using the *Goodall3* index. Finally the significant of the differences between the medians of these two distributions was determined using the Wilcox rank-sum test.
(TIF)

S6 Fig Replication of phenotypic convergence in patients without *de novo* CNVs. Overall, patients whose CNVs (inherited or of unknown inheritance) affect genes in the same pathway are phenotypically similar (*p* < 10^–40^). As per Fig. 4A The Y-axis gives the significance of the overall phenotypic similarity amongst patients with a specific phenotype whose variant genes belong to the associated pathways (A) or the extended pathway (B) with the phenotype (Intra) as compared to those patients with the phenotype without CNVs affecting genes in the pathway (Inter), with higher values indicating increasing relative similarity amongst association-contributing patients. Each point represents a single significant pathway-phenotype association, while the resources used to identify the pathways are shown on the X-axis (KEGG, MGI mouse KO phenotypes, GO, BS BrainSpan gene co-expression). Combinations of methods (e.g. GO-KEGG) illustrate the relative phenotypic similarity amongst patients possessing copy variant genes that individually contribute to multiple functional associations (see **Results**). “PPI” values are those among patients contributing the interacting molecular networks identified in **Fig. 2** (see **Results**). Dots coloured blue or red indicate nominally significantly phenotypic similarity or dissimilarity, respectively.
(TIF)

S7 Fig Phenotypic convergence of significant BrainSpan enrichments using different correlation thresholds. Edges in the BrainSpan co-expression network were restricted to those gene pairs with a correlation at least as high as each threshold. (A) Pearson correlation > = 0.6 (B) Pearson correlation > = 0.7 (C) Pearson correlation > = 0.8 (D) Pearson correlation > = 0.9. Dark bars are phenotypic convergence calculated using all patient phenotypes, light bars are phenotypic convergence calculated using only the child phenotypes of the original phenotype the enrichment was detected in.
(TIF)

S1 Table Click here for additional data file. Enrichments of genes whose 1:1 orthologues’ disruption in the mouse yields a particular phenotype, amongst the copy number variant genes of patients who share a particular phenotype (patient-phenotype group).
(XLSX)

S2 Table Click here for additional data file. Enrichments of GO terms amongst the copy number variant genes of patients who share a particular phenotype (patient-phenotype group).
(XLSX)

S3 Table Click here for additional data file. Enrichments of KEGG pathways amongst the copy number variant genes of patients who share a particular phenotype (patient-phenotype group).
(XLSX)

S4 Table Click here for additional data file. Enrichments among copy number variant genes in patients who share a particular phenotype (patient-phenotype group), of genes whose expression patterns are highly correlated.
(XLSX)

S5 Table Click here for additional data file. Genes that were individually identified by multiple functional enrichment/pathway approaches for a given patient-phenotype group (S1–S4 Tables).
(XLSX)

S6 Table Click here for additional data file. Genes that were individually identified by multiple functional enrichment/pathway approaches for a given patient-phenotype group whose protein products were found to cluster significantly in a protein-protein interaction network.
(XLSX)

S7 Table Click here for additional data file. Mappings between HPO and the LDDM ontologies for the PPI network phenotypes.
(XLSX)
