# Supplementary figures and images for: Gene Networks Underlying Convergent and Pleiotropic Phenotypes in a Large and Systematically-Phenotyped Cohort with Heterogeneous Developmental Disorders
Source: PLoS Genet. 2015 Mar 17;11(3):e1005012. doi: 10.1371/journal.pgen.1005012 (PMC4362763; doi:10.1371/journal.pgen.1005012)

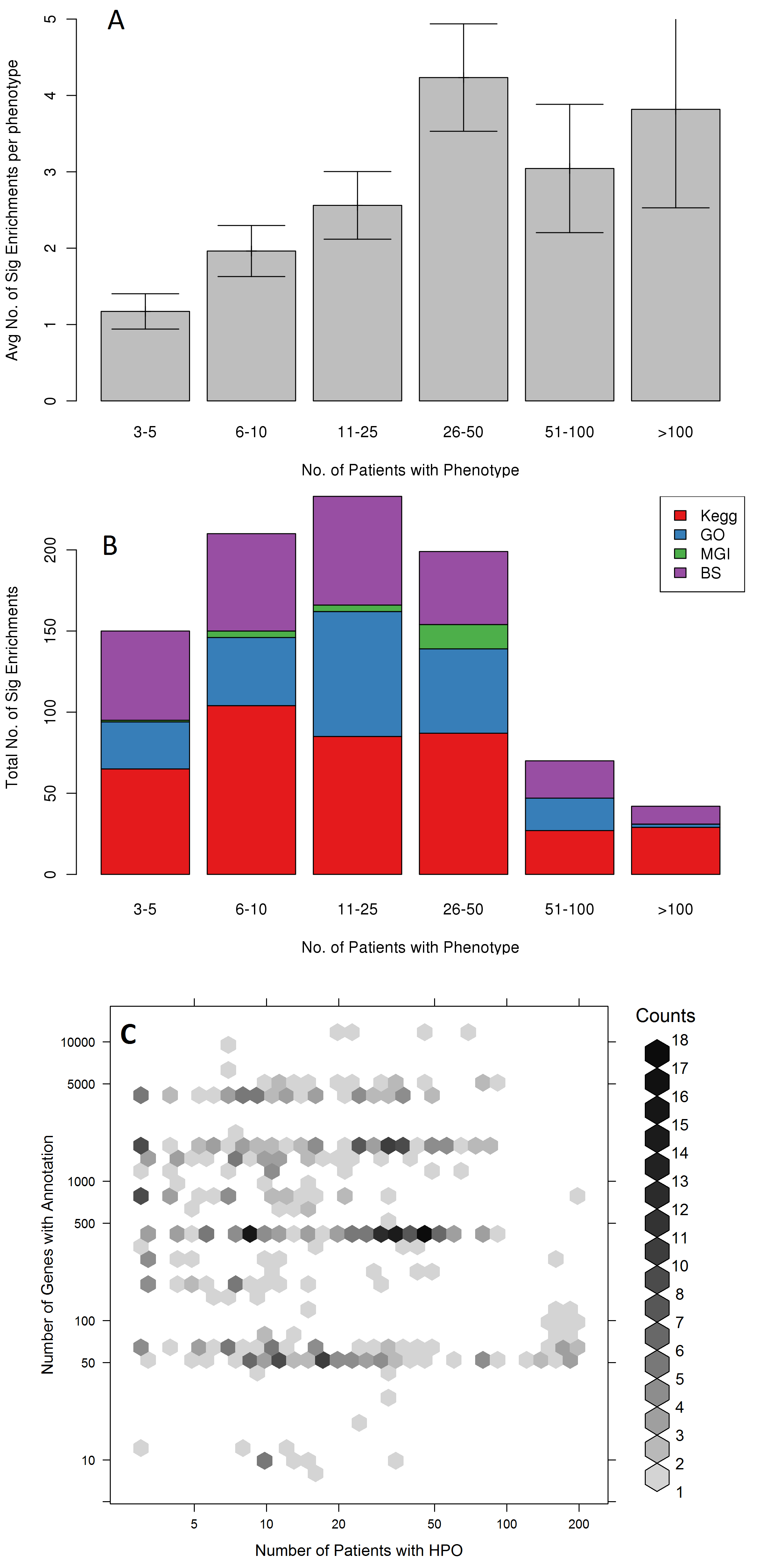

Supplement: S1 Fig — (A) Average number of significant functional enrichments per phenotype. Error bars indicate 95% Confidence Intervals. (B) Total number of significant enrichments by data type: Gene Ontology (GO), Kyoto Encyclopaedia of Genes and Genomes (KEGG), mouse knockout phenotypes (MGI), BrainSpan gene co-expression (BS). (C) Number of significant annotations (GO, KEGG, or MGI) vs the number of patients with the respective HPO and the number of genes in the whole genome with the respective annotation. (TIF) [file pgen.1005012.s001.tif]

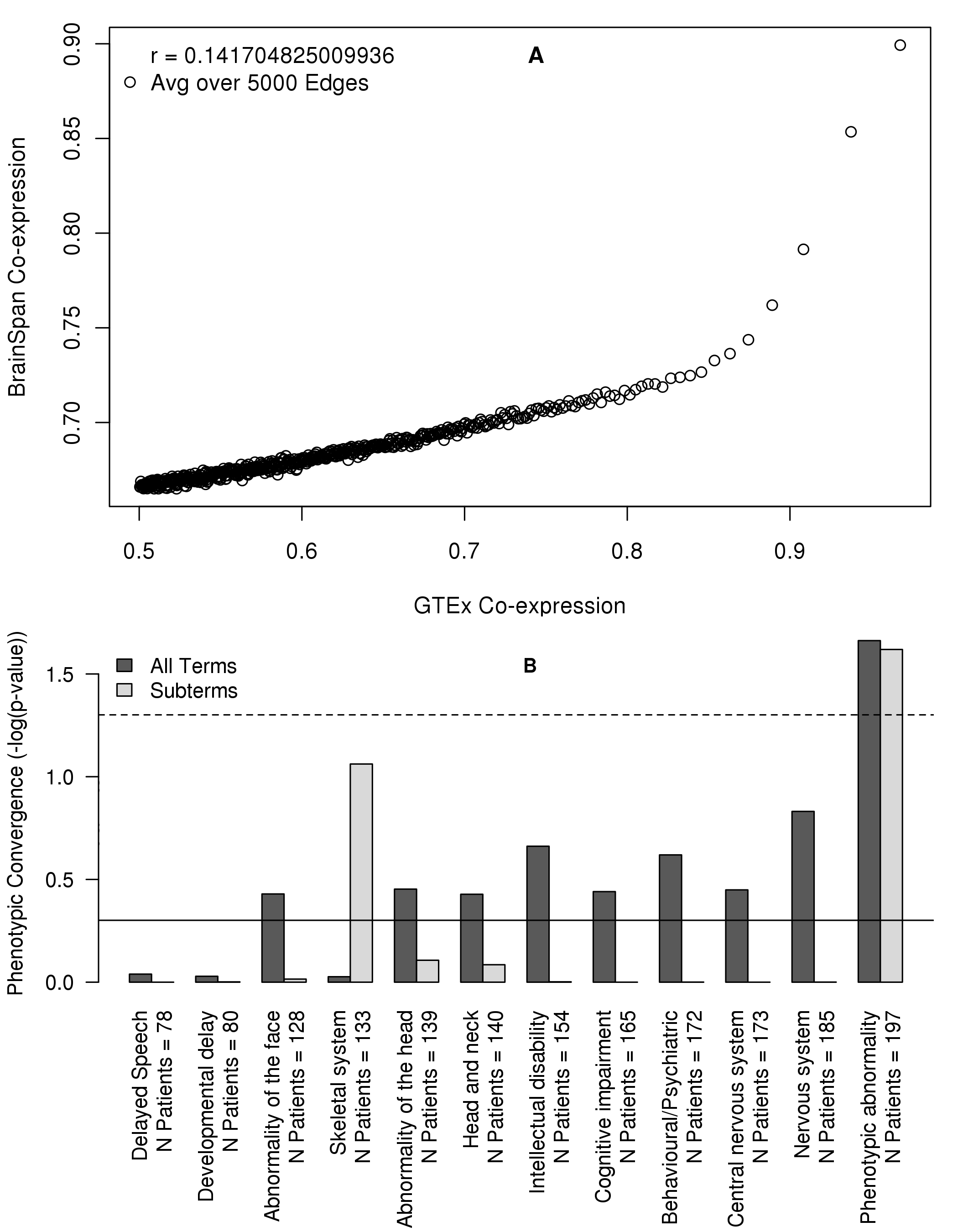

Supplement: S2 Fig — (A) Pearson correlation between GTEx and BrainSpan co-expression networks (all edges with r > 0.5 in both networks). Each point is the average taken over 5000 edges. The Pearson correlation coefficient on the unbinned data is noted in the corner of the plot (r = 0.14); (B) Phenotypic similarity of subset of patients contributing to a significant GTEx co-expression network. Solid line is p = 0.5, dashed line is p = 0.05. Dark bars are using all phenotypes to calculate phenotypic similarity; light bars are using only the subterms of the original human phenotype to calculate phenotypic similarity. (TIF) [file pgen.1005012.s002.tif]

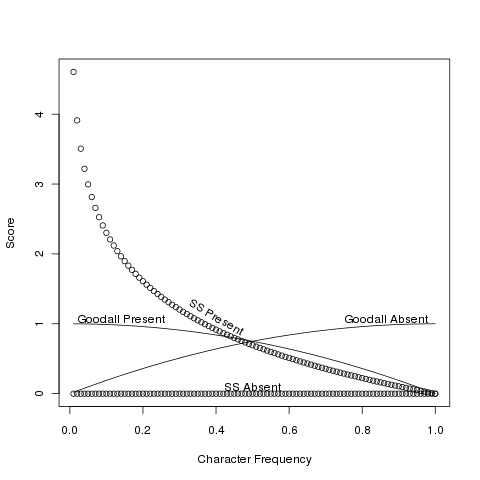

Supplement: S3 Fig — While semantic similarity strongly weights the presence of a shared rare character, nothing is learned from the shared absence of a character. By comparison, the Goodall metric considers both the presence of shared rare character and the absence of a common shared character towards the overall similarity. The Goodall metric is thus more suitable where both the presence and absence of phenotypes are known, as is the case here with the systematically phenotyped Nijmegen cohort. (TIF) [file pgen.1005012.s003.tif]

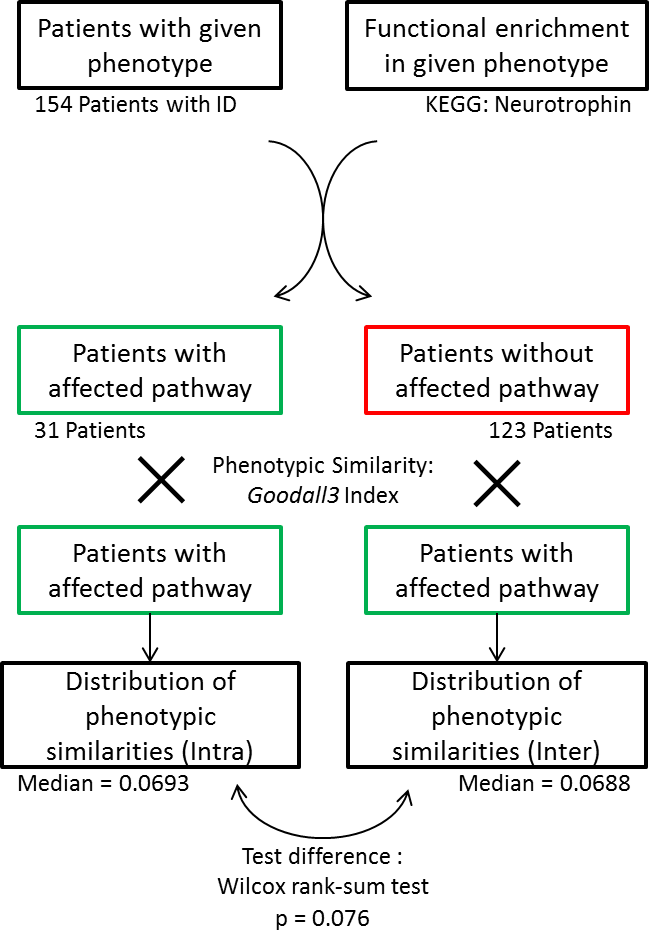

Supplement: S5 Fig — Each set of patients sharing a given phenotype was subdivided using each significant functional enrichment associated with that phenotype, eg. the KEGG Neurotrophin pathway, which was significantly enriched among CNV genes in patients with intellectual disability (ID). One group, ‘contributing patients’, contained all patients with the phenotype (ie. ID) whose CNV affected genes contributing to the functional enrichment (ie. belong to the Neurotrophin pathway); the other patients with the phenotype were placed in the ‘non-contributing’ group. Then the distributions of pair-wise patient phenotypic similarity within the ‘contributing’ group and between groups was calculated using the Goodall3 index. Finally the significant of the differences between the medians of these two distributions was determined using the Wilcox rank-sum test. (TIF) [file pgen.1005012.s005.tif]

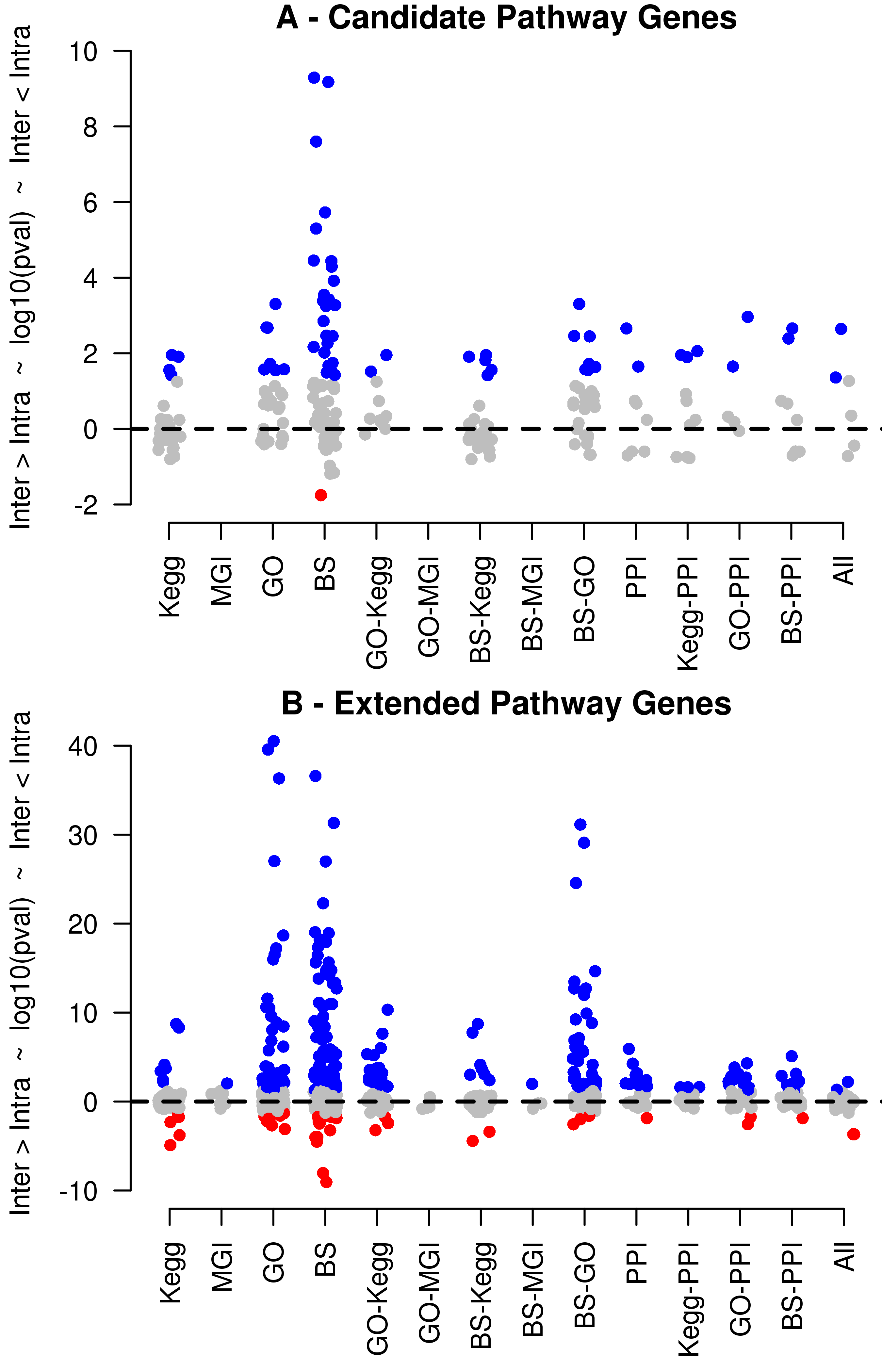

Supplement: S6 Fig — Overall, patients whose CNVs (inherited or of unknown inheritance) affect genes in the same pathway are phenotypically similar (p < 10–40). As per Fig. 4A The Y-axis gives the significance of the overall phenotypic similarity amongst patients with a specific phenotype whose variant genes belong to the associated pathways (A) or the extended pathway (B) with the phenotype (Intra) as compared to those patients with the phenotype without CNVs affecting genes in the pathway (Inter), with higher values indicating increasing relative similarity amongst association-contributing patients. Each point represents a single significant pathway-phenotype association, while the resources used to identify the pathways are shown on the X-axis (KEGG, MGI mouse KO phenotypes, GO, BS BrainSpan gene co-expression). Combinations of methods (e.g. GO-KEGG) illustrate the relative phenotypic similarity amongst patients possessing copy variant genes that individually contribute to multiple functional associations (see Results). “PPI” values are those among patients contributing the interacting molecular networks identified in Fig. 2 (see Results). Dots coloured blue or red indicate nominally significantly phenotypic similarity or dissimilarity, respectively. (TIF) [file pgen.1005012.s006.tif]

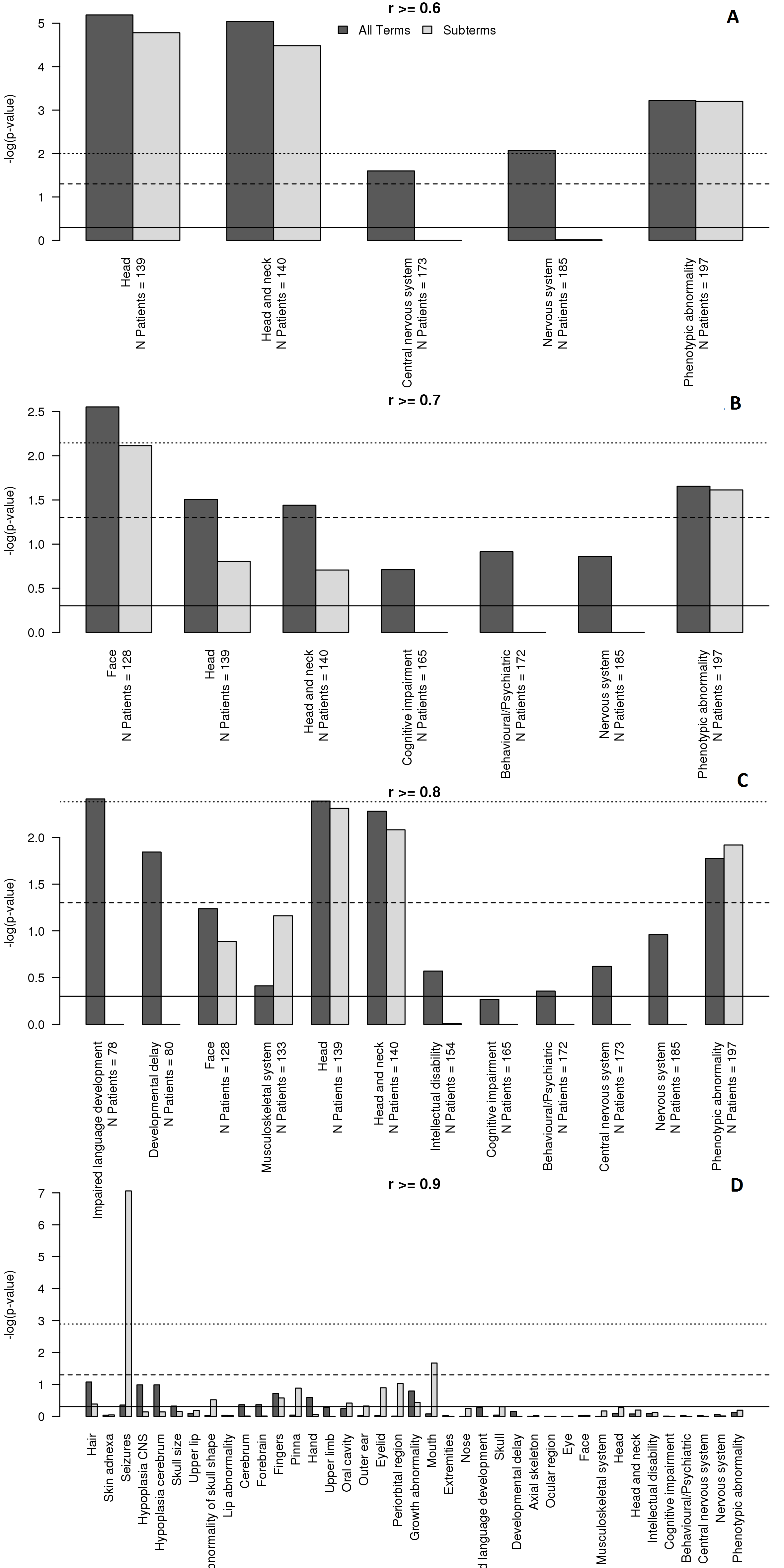

Supplement: S7 Fig — Edges in the BrainSpan co-expression network were restricted to those gene pairs with a correlation at least as high as each threshold. (A) Pearson correlation > = 0.6 (B) Pearson correlation > = 0.7 (C) Pearson correlation > = 0.8 (D) Pearson correlation > = 0.9. Dark bars are phenotypic convergence calculated using all patient phenotypes, light bars are phenotypic convergence calculated using only the child phenotypes of the original phenotype the enrichment was detected in. (TIF) [file pgen.1005012.s007.tif]
