# Supplementary material for: Gene Networks Underlying Convergent and Pleiotropic Phenotypes in a Large and Systematically-Phenotyped Cohort with Heterogeneous Developmental Disorders
Source: PLoS Genet. 2015 Mar 17;11(3):e1005012. doi: 10.1371/journal.pgen.1005012 (PMC4362763; doi:10.1371/journal.pgen.1005012)
Supplement: S4 Fig — The genes considered for these networks were those that were identified from multiple functional genomics/pathways approaches (GO, KEGG, BrainSpan, or MGI). See Fig. 2. Only those 14 PPI networks identified that contain a minimum of 5 genes are shown. (PDF) [file pgen.1005012.s004.pdf]

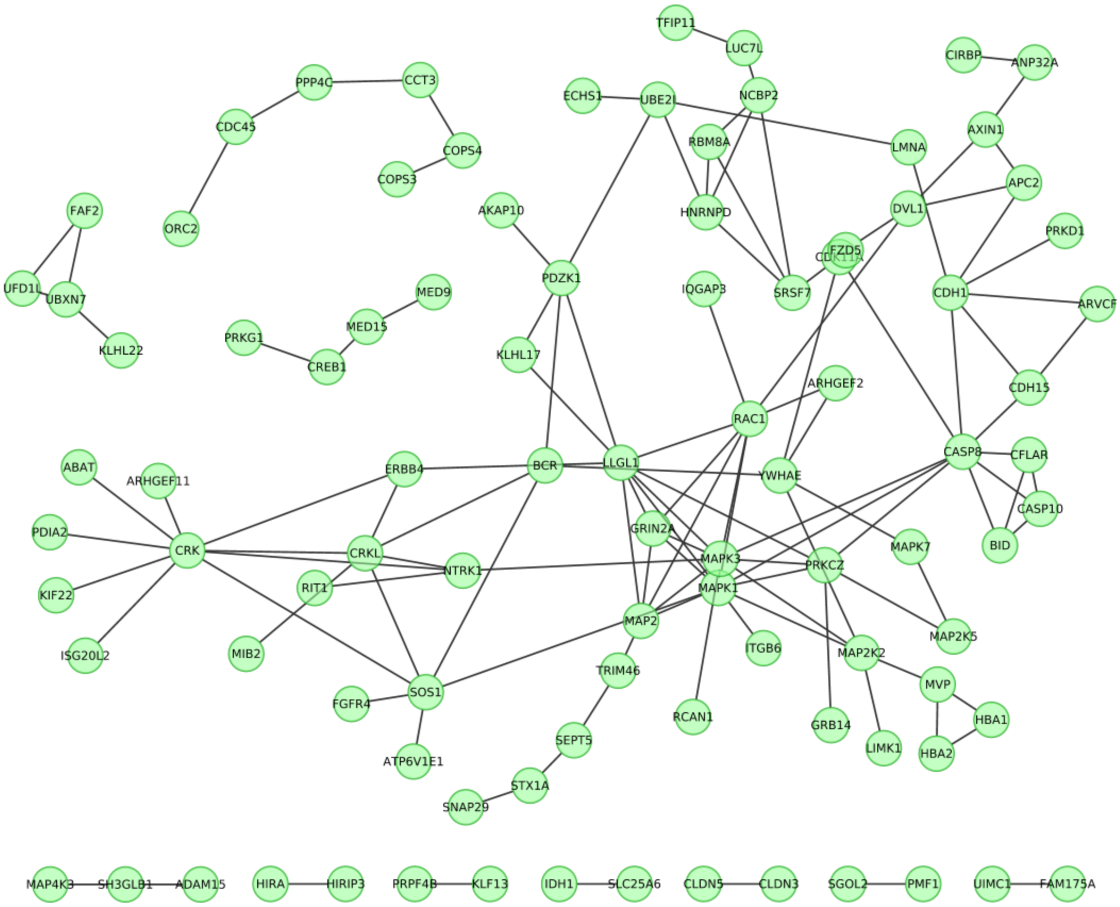

HP:0008050 ***Abnormality of the palpebral fissures,*** Largest Cluster: 66 genes

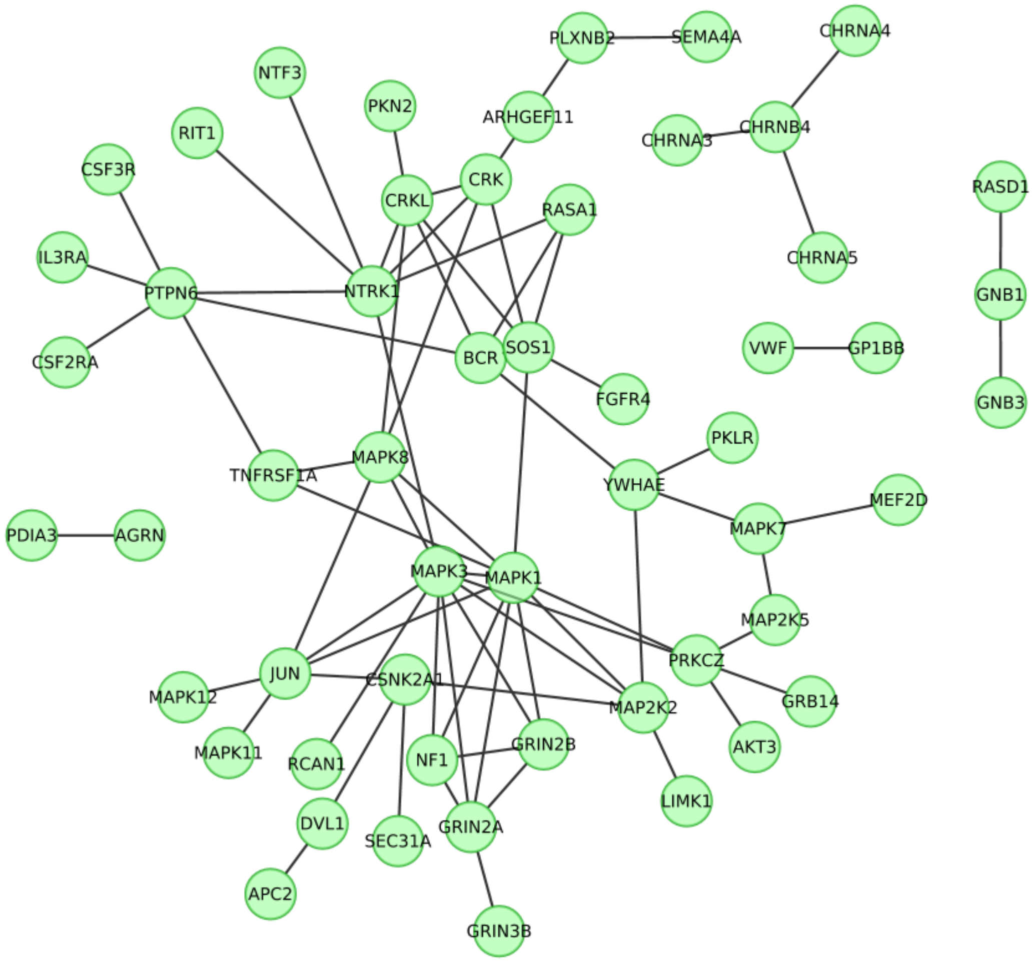

HP:0009121 ***Abnormality of the axial skeleton,*** Largest cluster: 43 genes

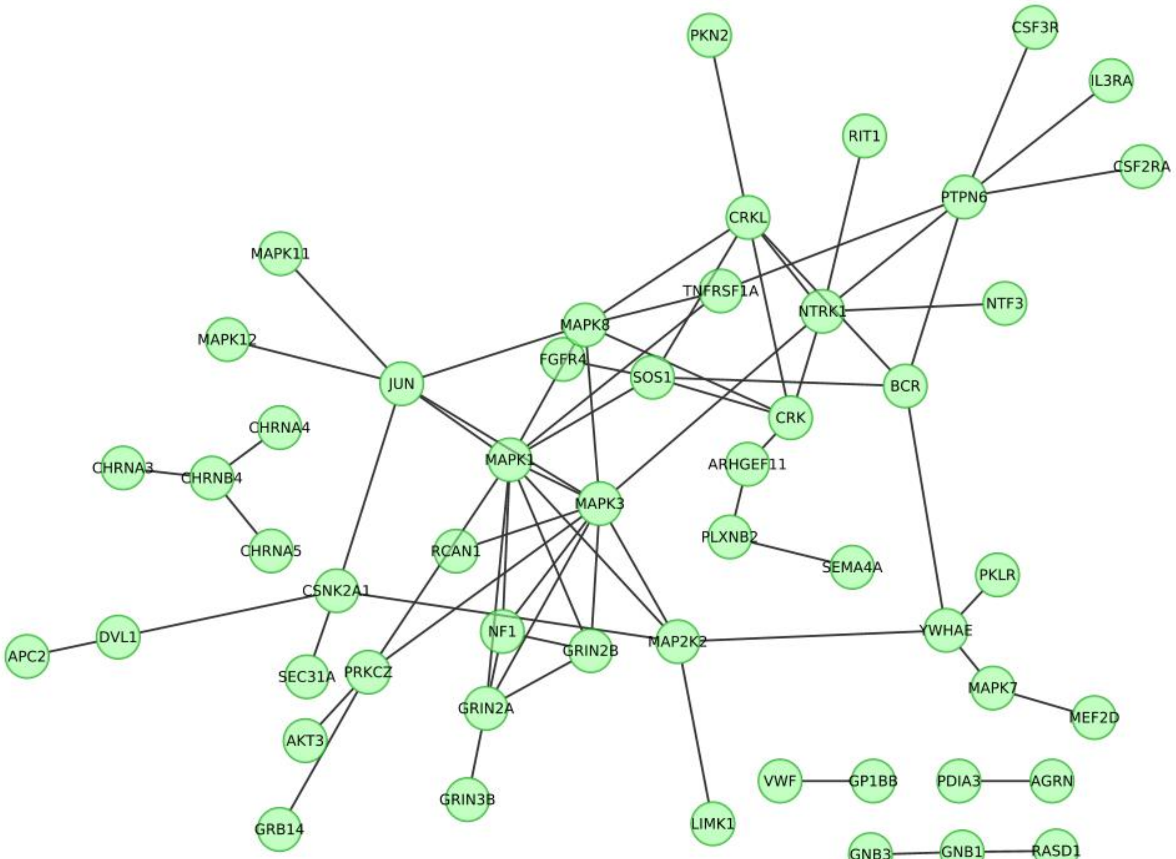

HP:000929 ***Abnormality of the skull,*** Largest cluster: 41 genes

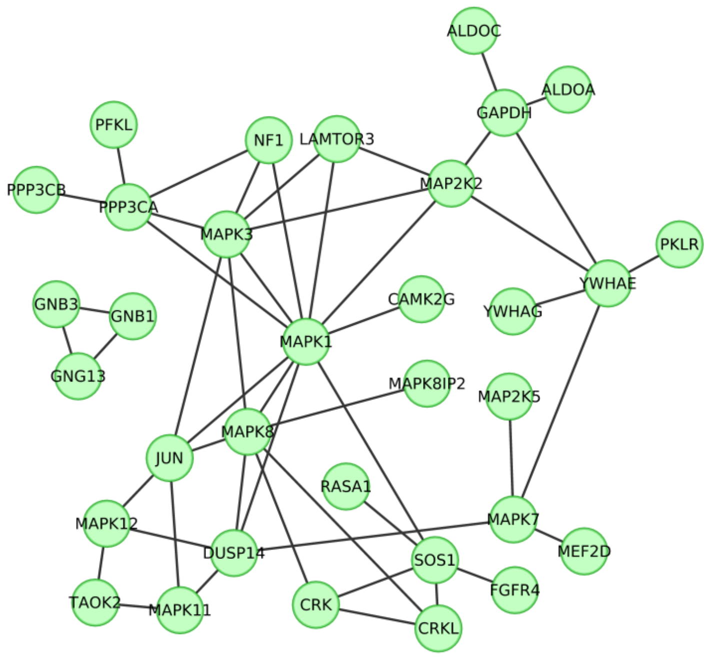

HP:0001249 ***Intellectual disability,*** Largest cluster: 30 genes

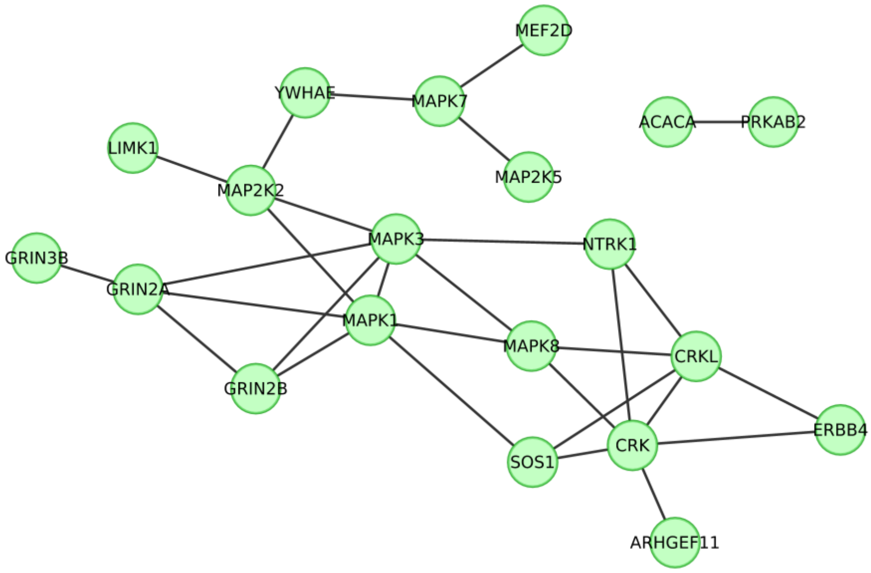

HP:0000163 ***Abnormality of the oral cavity,*** Largest cluster: 18 genes

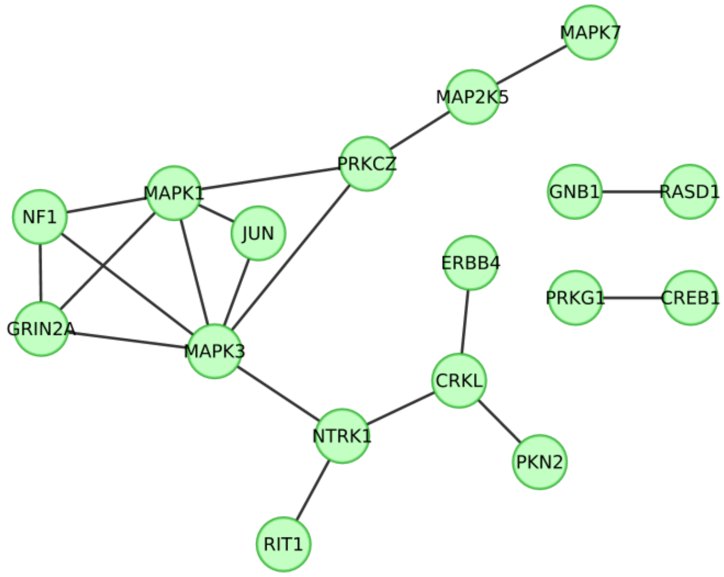

HP:0001438 ***Abnormality of the abdomen,*** Largest cluster: 13 genes

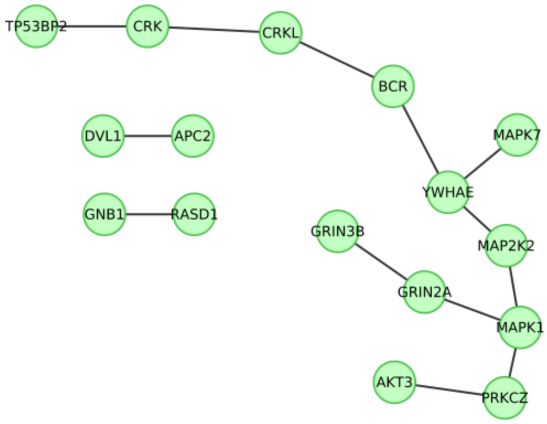

HP:0007364 ***Aplasia/Hypoplasia of the cerebrum,*** Largest cluster: 12 genes

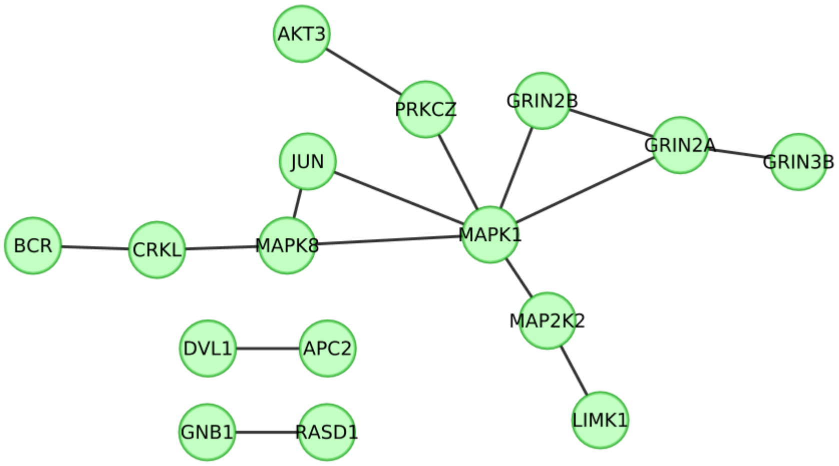

HP:0000240 ***Abnormality of skull size,*** Largest cluster: 12 genes

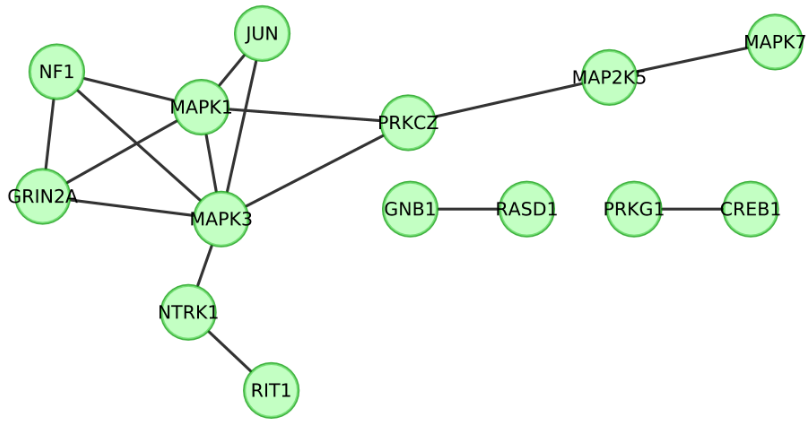

HP:0002012 ***Abnormality of the abdominal organs,*** Largest cluster: 10 genes

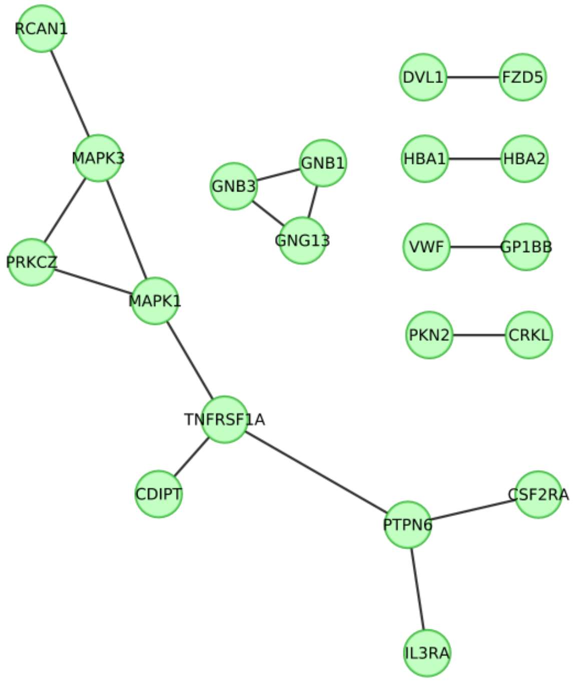

HP:0001999 ***Facial dysmorphism,*** Largest cluster: 9 genes

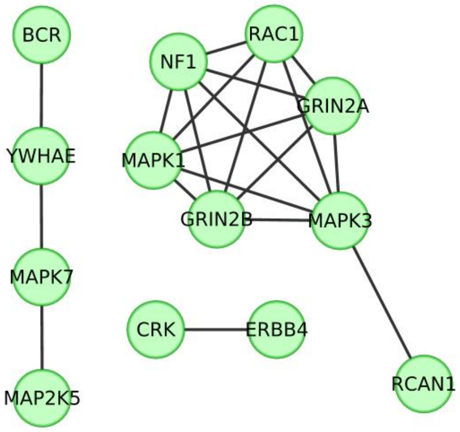

HP:0000209/HP:0000277 ***Abnormality of the mandible/jaws,*** Largest cluster: 7 genes

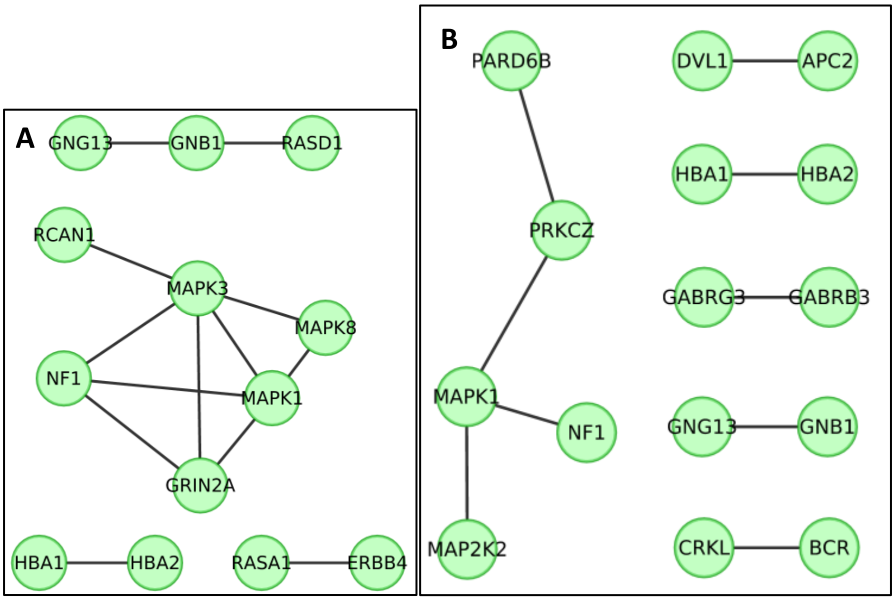

(A) HP:0000422 ***Abnormality of the nasal bridge,*** Largest cluster: 6 genes

(B) HP:0000119 ***Abnormality of the genitourinary system,*** Largest cluster: 5 genes

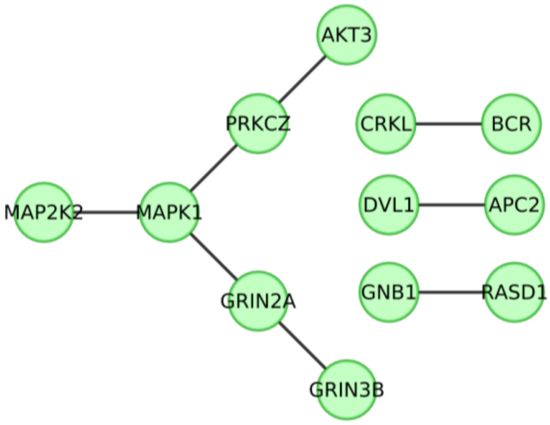

HP:0000252 ***Microcephaly,*** Largest cluster: 6 genes

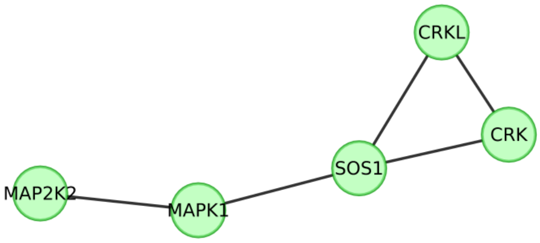

HP:0000164 ***Abnormality of the teeth,*** Largest cluster: 5 genes
